# Atorvastatin Added to Interferon Beta for Relapsing Multiple Sclerosis: 12-Month Treatment Extension of the Randomized Multicenter SWABIMS Trial

**DOI:** 10.1371/journal.pone.0086663

**Published:** 2014-01-30

**Authors:** Christian P. Kamm, Marwan El-Koussy, Sebastian Humpert, Oliver Findling, Yuliya Burren, Guido Schwegler, Filippo Donati, Martin Müller, Felix Müller, Johannes Slotboom, Ludwig Kappos, Yvonne Naegelin, Heinrich P. Mattle, for the SWABIMS Study Group

**Affiliations:** 1 Department of Neurology, Inselspital, University Hospital Bern and University of Bern, Bern, Switzerland; 2 Department of Diagnostic and Interventional Neuroradiology, Inselspital, University Hospital Bern and University of Bern, Bern, Switzerland; 3 Department of Neurology, Cantonal Hospital Aarau, Aarau, Switzerland; 4 Department of Neurology, Spitalzentrum Biel, Biel, Switzerland; 5 Department of Neurology, Cantonal Hospital Lucerne, Luzern, Switzerland; 6 Department of Neurology, Cantonal Hospital, Muensterlingen, Muensterlingen, Switzerland; 7 Department of Neurology, University Hospital, University of Basel, Basel, Switzerland; Postgraduate Medical Institute & Hull York Medical School, University of Hull, United Kingdom

## Abstract

**Background:**

Statins have anti-inflammatory and immunomodulatory properties in addition to lipid-lowering effects.

**Objectives:**

To report the 12-month extension of a phase II trial evaluating the efficacy, safety and tolerability of atorvastatin 40 mg/d added to interferon beta-1b (IFNB-1b) in relapsing-remitting multiple sclerosis (RRMS).

**Methods:**

In the randomized, multicenter, parallel-group, rater-blinded core study, 77 RRMS patients started IFNB-1b. At month three they were randomized 1∶1 to receive atorvastatin 40 mg/d or not in addition to IFNB-1b until month 15. In the subsequent extension study, patients continued with unchanged medication for another 12 months. Data at study end were compared to data at month three of the core study.

**Results:**

27 of 72 patients that finished the core study entered the extension study. 45 patients were lost mainly due to a safety analysis during the core study including a recruitment stop for the extension study. The primary end point, the proportion of patients with new lesions on T2-weighted images was equal in both groups (odds ratio 1.926; 95% CI 0.265–14.0007; p = 0.51). All secondary endpoints including number of new lesions and total lesion volume on T2-weighted images, total number of Gd-enhancing lesions on T1-weighted images, volume of grey and white matter, EDSS, MSFC, relapse rate, number of relapse-free patients and neutralizing antibodies did not show significant differences either. The combination therapy was well tolerated.

**Conclusions:**

Atorvastatin 40 mg/day in addition to IFNB-1b did not have any beneficial effects on RRMS compared to IFNB-1b monotherapy over a period of 24 months.

**Trial Registration:**

ClinicalTrials.gov NCT01111656

## Introduction

Statins have anti-inflammatory and immunomodulatory properties in addition to its cholesterol-lowering effects [1]. Various experimental studies suggest a positive effect of statins on multiple sclerosis (MS), a chronic inflammatory disorder of the central nervous system [2]–[6].

We therefore performed the SWiss Atorvastatin and Interferon Beta-1b trial in Multiple Sclerosis (SWABIMS), a multi-centre, randomized, parallel-group, rater-blinded study that evaluated the efficacy, safety and tolerability of atorvastatin 40 mg per os daily and subcutaneous interferon beta-1b (IFNB-1b) every other day compared to monotherapy with subcutaneous IFNB-1b every other day on relapsing-remitting MS (RRMS) over a period of 12 months [7], [8]. SWABIMS did not show any beneficial effect of atorvastatin added to IFNB-1b which is in line with other combination trials of statins and IFNB in RRMS (Table 1) [9]–[15].

**Table 1:** Overview of published clinical studies evaluating the combination of statins and IFNB in RRMS.

	Study type	Patients	Allocation	IFNB	Statin	Primary endpoint/Results
Paul F et al.2008 [9]	open-label baseline-to treatment trial	RRMS (n = 41)	IFNB+statin (n = 16); Statin (n = 25)	IFNB-1a 22 µg t.i.w. or IFNB-1b e.o.d.	atorvastatin80 mg/d	Trend towards reduction of Gd-enhancing lesions with IFNB+atorvastatin (p = 0.060)
Birnbaum G et al.2008 [11]	double-blind, placebo controlled trial	RRMS (n = 26)	IFNB+placebo (n = 9); IFNB+statin (n = 17)	IFNB-1a 44 µg t.i.w.	atorvastatin 80mg/d (n = 10) or40 mg (n = 7)	Increased MRI and clinical disease activity with atorvastatin (p = 0.019)
Rudick RA et al.2009 [12]	post hoc analysis	RRMS (n = 582)	IFNB (n = 542); IFNB+statin (n = 40)	IFNB-1a 30 µg once weekly	atrovastatin or simvastatin	No difference in annualized relapse rate and secondary endpoints
Lanzillo R et al.2010 [13]	Longitudinal controlled trial	RRMS (n = 45)	IFNB (n = 24); IFNB+statin (n = 21)	IFNB-1a 44 µg s.c. t.i.w.	atorvastatin20 mg/d	Fewer Gd− enhancing lesions versus baseline (p = 0.007) and fewer relapses versus the two pre randomization years (p<0.001) with atorvastatin
Togha M et al.2010 [10]	double-blind randomized controlled trial	RRMS (n = 80)	IFNB+placebo (n = 38); IFNB+simvastatin (n = 42)	IFNB-1a 30 µg once weekly	simvastatin40 mg/d	Lower relapse rate with simvastatin (p = 0.01)
Sörensen PS et al.2011 [14]	placebo-controlled randomised trial	RRMS (n = 307)	IFNB+statin (n = 151); IFNB+placebo(n = 156)	IFNB-1a 30 µg once weekly	simvastatin80 mg/d	No difference in annualized relapse rate and secondary endpoints
SWABIMS [8]	randomized controlled trial	RRMS (n = 76)	IFNB+statin (n = 38); IFNB (n = 38)	IFNB-1b e.o.d.	atorvastatin40 mg/d	No difference of patients with new T2-lesions and in secondary endpoints
SWABIMSExtensionStudy	randomized controlled trial	RRMS (n = 27)	IFNB+statin (n = 13); IFNB (n = 14)	IFNB-1b e.o.d.	atorvastatin40 mg/d	No difference of patients with new T2-lesions and in secondary endpoints

n, number; IFNB, interferon beta; t.i.w., three times per week; d, day; e.o.d., every other day.

Herein, we present the results of the preplanned extension of SWABIMS for another 12-months with unchanged medication that was designed to test the effect of atorvastatin 40 mg in addition to IFNB-1b compared to IFNB-1b monotherapy over a period of 24 months (SWABIMS Extension Study).

## Materials and Methods

### Patients

In the core study, patients with RRMS according to the 2005 McDonald’s criteria, disease duration>three months, at least one relapse in the past two years, ≥ three lesions on spinal or brain-MR or both, baseline Expanded Disability Status Scale (EDSS) score from 0 to 3.5 (inclusive), and age from 18 to 55 years were eligible to participate [16], [17]. Main exclusion criteria were primary or secondary progressive MS, clinically isolated syndrome, previous therapy with monoclonal antibodies, mitoxantrone, other cytotoxic or immunosuppressive drugs, and IFNB or glatiramer acetate within the last 12 months. All patients who completed the core study were eligible to enter the extension study.

### Ethics Statement

Each patient had to provide a separate written informed consents prior to the extension study and the study was conducted in accordance with the International Conference on Harmonisation Guidelines for Good Clinical Practice (1996) and the Declaration of Helsinki (2006), and approved by the responsible cantonal ethics committees of the participating centers (Cantonal ethics committee Bern: University hospital Bern, Spitalzentrum Biel; Cantonal ethics committee Aarau: Cantonal Hospital Aarau; Cantonal ethics committee Luzern: Cantonal Hospital Lucerne; Cantonal ethics committee Thurgau: Cantonal Hospital Muensterlingen; Cantonal ethics committee Basel: University Hospital Basel) and the “Swiss agency for the authorisation and supervision of therapeutic products” (Swissmedic) [18], [19]. The trial Registration Identifier for the SWABIMS Extension Study is NCT01111656 (clinicaltrials.gov). The trial Registration Identifier of the core study (SWABIMS) is NCT00942591 (clinicaltrials.gov).

### Study Design

The protocol for this trial and supporting CONSORT checklist are available as supporting information; see Checklist S1 and Protocol S1. The core study was a multi-centre, randomized, parallel-group, rater-blinded study conducted in eight Swiss hospitals [7], [8]. At the beginning of the study, all patients started IFNB-1b (Betaferon®/Betaseron®, Bayer Schering Pharma) for three months [20]. At month three, they were randomized 1∶1 to receive atorvastatin 40 mg/day or not in addition to IFNB-1b for another 12 months.

Randomization was performed centrally by the clinical research organisation (CRO) after baseline visit in four-block size, according to the randomization list (atorvastatin “yes” or “no”) generated with “RANCODE Professional 3.6″ [21]. In the extension study, patients continued with unchanged medication for another 12 months. The last visit of the core study was the baseline visit of the extension study ( = month 15).

In both studies, patients and treating physicians were aware, whether atorvastatin was added. Placebo was not dispensed. Examining physicians scoring disability (EDSS; Multiple Sclerosis Functional Composite [MSFC]) and neuroradiologists evaluating magnetic resonance images (MR) were blinded to treatment assignments [22].

### Study Endpoints

The study endpoints of the core and the extension study were identical. Data at the end of the extension study were primarily compared to data at month three of the core study, before randomization to atorvastatin or not.

The primary endpoint was the proportion of patients with new lesions on T2-weighted MR images. Secondary endpoints were the number of new lesions on T2-weighted images, change in total lesion volume on T2-weighted images (burden of disease), total number of gadolinium (Gd−) enhancing lesions on T1-weighted images, changes in volume of grey and white matter, clinical disease progression (EDSS, MSFC), relapse rate, time to first relapse, number of relapse-free patients, and neutralizing antibodies (NAbs).

Adverse events (AE), laboratory data, vital signs and concomitant medication were analyzed as safety variables.

### Study Procedures

Regular visits were conducted at baseline, month six and month 12 during the extension study. Safety assessments including laboratory analysis and evaluation of concomitant medication were performed on each visit. At baseline and month 12, the EDSS, MSFC, neutralizing antibodies (NAbs), brain MRI, as well as other efficacy and safety endpoints were aditionally performed. Furthermore, patients were called at month three and nine for the assessment of safety issues and concomitant medication.

Atorvastatin use was controlled by counting the returned tablets at month 6 and 12. A patient was considered compliant when he took at least 80% of all atorvastatin tablets.

A relapse was defined as a newly appearing objective neurological abnormality in the absence of fever or known infection, correlating with the patient’s reported symptoms, lasting for at least 24 hours, occuring at least 30 days after a preceding clinical event, and increasing the total EDSS score or at least one of the functional systems of the EDSS score. Fatigue, mental and/or vegetative symptoms were not classified as relapse. Relapses were treated within seven days with intravenous methylprednisolone 500 mg/day for five days followed by tapering-out with oral prednisolone.

Atorvastatin was reduced to 20 mg/d in case of a more than threefold increase of transaminases and stopped in case of more than fivefold increase. Afterwards, liver enzymes were controlled regularly and atorvastatin was continued when transaminases were below a threefold increase.

MR scans were acquired at each hospital on 1.5 Tesla scanners at baseline and month 12. The MR protocol included T1-weighted axial spin-echo, T1-weighted sagittal 3D MPRAGE, axial dual-echo i.e. proton-density, T2-weighted turbo-spin-echo images and axial T1-weighted spin-echo images after intravenous Gd-injection.

MR scans were assessed centrally by two neuroradiologists at the Department of Diagnostic and Interventional Neuroradiology, Inselspital, University Hospital Bern [23], [24]. A T2 lesion was defined as an area of increased signal on both the proton-density and the T2-weighted images. Disagreeing interpretations were discussed among the neuroradiologists to reach consensus. The image processing was performed with an algorithm enabling semi-automatic volumetry [25].

Laboratory analyses except NAbs were performed by Viollier AG (4002 Basel, Switzerland). NAbs were assessed at the Ospedale San Luigi, Orbassano, Italy. The cytopathic effect assay was used as recommended by the World Health Organization [26]. Data from the neutralisation assay were reported as reciprocal of the highest dilution of serum inducing 50% neutralisation. The neutralisation titre was calculated according to Kawade’s formula and expressed in laboratory units (LU). A concentration of >20 LU/ml was considered positive [27]. Patients with one or more NAb positive titers were defined NAb positive.

### Statistical Analysis

SAS version 9.2 was used for all statistical analyses. For the core study with regard to the first treatment year, a sample size of 38 patients in each group was needed to obtain a power of 84% to detect the difference between the monotherapy group proportion, π1, of 0.610 and the combination therapy group proportion, π2, of 0.910 with a 0.05 two-sided significance level in the Fisher’s exact test [8]. All patients, who took at least one dose of study medication and had at least one follow-up observation were analyzed (Full Analysis Set [FAS]). Missing values were treated as missing, exept for severity and relationship of adverse events to study drugs. If severity or relationship was missing, the adverse event was regarded as severe or related to the study drug respectively.

Categorical data were described by frequency and percentage, continuous data by mean, standard deviation, minimum, 1^st^ quartile, median, 3^rd^ quartile and maximum. Hypothesis tests were carried out with a α-level of 0.05, two-sided. All inferential analyses were presented by p-values, point estimations and two-sided 95% CI for treatment differences. If the assumption of normality in the linear models was not fulfilled, transformations of the data or non-parametric approaches like the Wilcoxon signed rank test were used.

Differences between treatment groups at baseline were tested using t-test or Fisher’s exact test depending on the distribution of the data.

The primary efficacy variable was the proportion of patients with new T2 lesions at month 12 of the SWABIMS Extension Study compared to month three of SWABIMS, i.e. new T2 lesions emerging over a period of 24 months with active treatment. Based on a logistic regression model with the factors treatment and gender and the covariates number of T2 lesions, number of Gd-enhancing lesions, EDSS, relapse rate and time since MS diagnosis at baseline at month three, the two-sided hypothesis of equality between the two treatments was tested at an α-level of 0.05. The results were presented as odds ratios and the associated two-sided 95% CI and p-values. Furthermore, a Fisher’s exact test for proportions was executed to test for the unadjusted treatment effect.

Secondary efficacy variables were analyzed with covariance, logistic regression models or Fisher’s exact test depending on the distribution. Time to first relapse was analyzed with non-parametric methods for failure time data (Wilcoxon test).

Assessments of safety and tolerability variables were presented by treatment group. AEs were summarized for each treatment group by presenting the number and percentage of subjects having an event, the number and percentage of event in each system organ class and preferred term, as well as severity and relationship to the study drug.

Any medication taken during the study was classified as concomitant and coded using WHO-Drug 2007.1.

## Results

**Figure 1:**
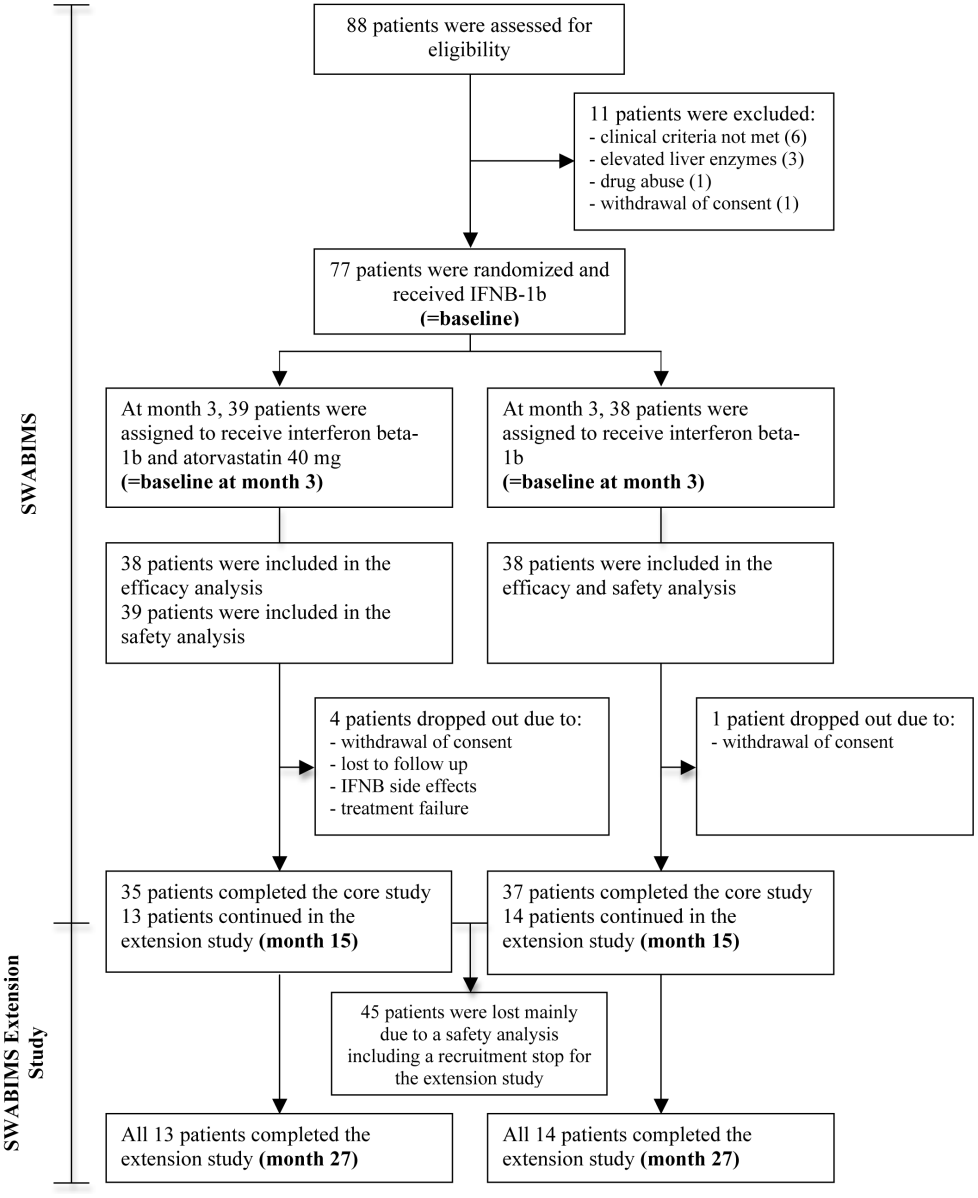
CONSORT Flow diagram showing enrollment, allocation and follow-up of patients in the SWABIMS and SWABIMS Extension Study.

From August 2006 to March 2009, 27 out of 72 patients who finished the core study entered the extension study. 45 patients were lost mainly due to a safety analysis during the core study including a recruitment stop for the extension study imposed by the local institutional review board (IRB), because a study of Birnbaum et al. suggested a negative effect of statins in combination with IFNB in MS [11]. Furthermore, two of the eight centres of the core study did not participate in the extension study. The IRB decision was revised after a safety analysis performed by B. Weinshenker and M. Matiello (Rochester MN, USA) showed no reason to terminate the trials which led to a continuation of the study. 14 patients were in the IFNB-1b group and 13 patients in the atorvastatin/IFNB-1b group. All 27 patients completed the extension study (Figure 1). Because of the unintended small patient number we performed a new power analysis that showed that a sample size of 13 in each group is needed to obtain power of 81% to detect differences between the atorvastatin/IFNB-1b group proportion, π1, of 0,923 and the IFNB-1b group proportion, π2, of 0,373 with a 0,050 two-sided significance level in the Fisher’s exact test with regard to the overall treatment duration of 24 month [8].

The atorvastatin compliance was >80% during the study and all relapses were treated with steroids as defined above.

The baseline demographic charateristics and the disease characteristics at month three of the core study, before randomisation to atorvastatin or not, showed no significant differences regarding the treatment groups (Table 2).

**Table 2:** Patient characteristics at screening/baseline of month three of SWABIMS.

Characteristics	Atorvastatin/Interferon beta-1b	Interferon beta-1b	P Value
	N = 13	N = 14	
**Demographic characteristics at screening/baseline**			
**Age (years)**			
Mean ± SD	32.15±9.61	36.93±8.24	0.17
Median (range)	30 (19–50)	39 (18–46)	
**Gender (N, %)**			
Male	3 (23.1%)	5 (35.7%)	
Female	10 (76.9%)	9 (64.3%)	0.68
**Caucasian (N, %)**	13 (100%)	14 (100%)	
**Height (cm)**			
Mean ± SD	171.08±7.61	170.71±10.63	
Median (range)	170 (156–188)	168 (157–187)	0.77
**Weight (kg)**			
Mean ± SD	69.19±11.29	77.46±22.06	
Median (range)	68 (54.6–100)	73.5 (52–124)	0.47
**BMI (kg/m2)**			
Mean ± SD	23.82±4.62	26.45±7.01	
Median (range)	24.68 (18.14–34.6)	24.45 (20.06–45.55)	0.51
**MR findings at month 3 of SWABIMS**			
**No. of T2 hyperintense lesions**			
N	12	14	
Mean ± SD	26.17±23.03	19.86±16.38	
Median (range)	21.5 (6–92)	13.5 (2–58)	0.46
**Total volume of T2 hyperintense lesions [cm^3^]**			
N	12	14	
Mean ± SD	3.6±3.31	2.93±2.98	
Median (range)	2.96 (0.36–12.35)	1.78 (0.15–8.98)	0.40
**No. of GD-enhancing lesions on T1-weighted images**			
N	13	14	
Mean ± SD	0.23±0.6	0.0±0.0	
Median (range)	0 (0–2)	0 (0–0)	0.15
**Total volume of GD-enhancing lesions on T1-weighted images [cm^3^]**			
N	13	14	
Mean ± SD	0.03±0.08	0±0	
Median (range)	0 (0–0.3)	0 (0–0)	0.15
**Volume of grey matter [cm^3^]**			
N	9	13	
Mean ± SD	728.11±74.4	736.65±84.99	
Median (range)	729.4 (620–867)	732.3 (614–880)	0.95
**Volume of white matter [cm^3^]**			
N	9	13	
Mean ± SD	432.73±41.58	435.55±78.13	
Median (range)	423.5 (386–494)	450.9 (270–544)	0.95
**Volume of grey and white matter [cm^3^]**			
N	9	13	
Mean ± SD	1160.86±84.61	1172.20±127.43	
Median (range)	1163.0 (1070–1336)	1149.8 (991–1379)	0.89
**Clinical characteristics**			
**MS duration at screening (years)**			
N	13	14	
Mean ± SD	1.45±4.51	1.21±2.2	0.45
**No. of relapses in the past 2 years before screening (N, %)**			
N	13	14	
1	6 (46.2%)	5 (35.7%)	
2	7 (53.8%)	9 (64.3%)	
3	0 (0%)	0 (0%)	
4	0 (0%)	0 (0%)	
8	0 (0%)	0 (0%)	0.70
**EDSS at month 3**			
N	13	14	
Mean ± SD	1.88±0.79	1.75±0.91	
Median (range)	2 (1–3.5)	2 (0–3)	0.96
**MSFC at month 3**			
N	13	14	
Mean ± SD	0.42±0.26	0.24±0.39	
Median (range)	0.41 (−0.05–0.8)	0.37 (−0.37–0.73)	0.32

N: number of patients; SD: standard deviation; EDSS: Expanded Disability Status Scale; MSFC: Multiple Sclerosis Functional Composite; BMI: body mass index; ns: no significant difference.

The results for the primary and secondary efficacy endpoints are given in Table 3.

**Table 3:** Efficacy endpoints: Month 3 of SWABIMS compared to the end of SWABIMS Extension Study (month 27) (FAS, N = 27).

Endpoint	Atorvastatin/Interferon-beta-1b	Interferon-beta-1b	P Value
	N = 13	N = 14	
**MR endpoints**			
**Proportion of patients with new lesions on T2-weighted images, month 3 to month 27 (N; %)**			
N	12	14	
Yes	8 (66.67)	7 (50.00)	
No	4 (30.33)	7 (50.00)	
Odds ratio for atorvastatin/IFNB-1b vs. IFNB-1b (95% CI)	1.926 (0.265 to 14.007)		0.51
**No. of new lesions on T2-weighted images, month 3 to month 27**			
N	12	14	
Mean ± SD	5.0±6.59	1.4±1.82	
Median (range)	1 (0–16)	0.5 (0–5)	
Treatment difference for atorvastatin/IFNB-1b vs. IFNB-1b (95% CI)¶	3.64 (−0.37 to 7.65)		0.07
**Change in lesion volume [cm^3^] on T2-weighted images, month 3 to month 27**			
N	12	13	
Mean ± SD	−0.0±1.51	−0.6±1.17	
Median (range)	0.3 (−3–3)	−0.4 (−3–0)	
Treatment difference for atorvastatin/IFNB-1b vs. IFNB-1b (95% CI)¶	0.55 (−0.35 to 1.44)		0.22
**Total number of Gd-enhancing T1-lesions, month 3 to month 27**			
N	13	14	
Mean ± SD	−0.1±0.76	0.0±0.00	
Median	0 (−2−1)	0 (0−0)	
Treatment difference for atorvastatin/IFNB-1b vs. IFNB-1b (95% CI)¶	0.22 (−0.03 to 0.48)		0.08
**Change of grey matter volume [cm^3^], month 3 to month 27**			
N	8	11	
Mean ± SD	3.9±35.85	−18.3±61.23	
Median (range)	−7.7 (−38–73)	−5.2 (−190–45)	0.72
**Change of white matter volume [cm^3^], baseline at month 3 to month 27**			
N	8	11	
Mean ± SD	−0.8±18.34	8.6±42.02	
Median (range)	0.7 (−31–30)	−1.7 (−42–118)	0.81
**Change of grey and white matter volume [cm^3^], month 3 to month 27**			
N	8	11	
Mean ± SD	3.1±30.22	−9.7±42.09	
Median (range)	−0.3 (−38–47)	−11.7 (−79–67)	0.82
**Clinical endpoints**			
**Change in EDSS score, month 3 to month 27**			
N	13	14	
Mean ± SD	0.154±1.2142	−0.036±1.1174	
Median (range)	0 (−2–3.50)	0 (−2–2)	
Least squares means for effect treatment (95% CI)¶	0.66 (−0.25 to 1.56)		0.14
**Change in MSFC score, month 3 to month 27**			
N	13	14	
Mean ± SD	−0.3±0.62	−0.4±0.53	
Median (range)	−0.2 (−1–1)	−0.2 (−2–0)	
Least squares means for effect treatment (95% CI)¶	0.07 (−0.41 to 0.56)		0.74
**Relapses, month 3 to month 27**			
N	13	14	
Relapse-free (N, %)			
No	7 (53.85)	4 (28.57)	
Yes	6 (46.15)	10 (71.43)	
Odds ratio of atorvastatin/IFNB-1b vs. IFNB-1b (95% CI) for “Patient is relapse-free”)	0.386 (0.054 to 2.743)		0.34
No. of relapses			
Total number	14	5	
Mean ± SD	1.1±1.44	0.4±0.63	
Median (range)	1.0 (0–4)	0.0 (0–2)	0.23
**Neutralizing antibodies (NAb)**			
NAb-positive (N, %)			
N	11	12	
No	3 (27.27)	6 (50)	
Yes	8 (72.73)	6 (50)	
Odds ratio of atorvastatin/IFNB-1b vs. IFNB-1b (95% CI) for “patient is NAb-positive”	4.10 (0.37 to 44.89)		0.25
Change from NAb-positive to NAb-negative			
N	10	9	
No	7 (70)	8 (88.89)	
Yes	3 (30)	1 (11.11)	
Odds ratio of atorvastatin/IFNB-1b vs. IFNB-1b (95% CI) for “patient is NAb-positive”	Cannot be calculated due to low N		0.21

N: number of patients with data; SD: standard deviation; EDSS: Expanded Disability Status Scale; MSFC: Multiple Sclerosis functional Composite; ¶ Treatment differences were calculated using ANCOVA.

The proportion of patients with new lesions on T2-weighted images showed no differences according to the logistic regression model (p = 0.51). The adjusted odds ratio (OR) and the 95% CI for the treatment difference of atorvastatin/IFNB-1b vs. IFNB-1b were 1.926 and 0.265 to 14.0007. To test the unadjusted treatment differences, an exploratory analysis with Fisher’s exact test was performed. Again, no significant difference was detected (p = 0.45).

The predefined secondary endpoints number of new lesions and total lesion volume on T2-weighted images, total number of Gd-enhancing lesions on T1-weighted images, volume of grey and white matter, EDSS, MSFC (including subscores), relapse rate, number of relapse-free patients and NAb did not show any significant differences between the treatment groups as well. The secondary endpoint time to next relapse was not calculated due to a low number of events.

Some data on MR endpoints were missing due to movement artefacts during single MR sequences. Furthermore, two centers did not provide adequate MRI data for grey and white matter analysis and did not collect NAbs explaining the lower numbers of individuals in these endpoints.

There was a trend towards a higher number of new lesions on T2-weighted images and total number of Gd-enhancing lesions on T1-weighted images in the atorvastatin/IFNB-1b group.

An ANCOVA model with new lesions on T2-weighted images respectively the total number of Gd-enhancing lesions on T1-weighted images as dependent variables and new lesions on T2-weighted images, total number of Gd-enhancing lesions on T1-weighted images, EDSS, relapse rate, gender, disease duration and treatment as independent variables showed that the number of Gd-enhancing lesions at baseline respectively gender had a relevant influence on these enpoints whereas treatment did not.

Details on AEs by system organ class are given in Table 4. During the extension study any AEs including serious and severe AEs occurred equally in both groups. There was no discontinuation of the study drug due to AEs. Two AEs, an influenza-like illness and increased hepatic enzymes were judged as related to the study drug in the atorvastatin/IFNB-1b group. Elevated liver enzymes occurred in two patients of the atorvastatin/IFNB-1b group without clinical relevance. In the atorvastatin/IFNB-1b group, all AEs were classified as mild or moderate. There was no severe or serious AE. In the IFNB-1b group, AEs were classified as mild or moderate except for one patients with a foot fracture that was classified as a severe as well as serious AE.

**Table 4:** Adverse events SWABIMS Extension Study by system organ class MedDRA (FAS, N = 27).

Events N (%)	Atorvastatin/Interferon-beta-1b	Interferon-beta-1b
	(N = 13)	(N = 14)
**Total number of adverse events**	7	7
**Adverse events (AE) by number of subjects**		
Overall adverse event	15 (53.8%)	12 (50.0%)
**Subjects with**		
any AE	7 (53.8%)	7 (50.0%)
any serious AE	0 (0%)	1 (7.1%)
any severe AE	0 (0%)	1 (7.1%)
any AE related to study drug	2 (15.4%)	0 (0.0%)
any AE leading to discontinuation of study drug	0 (0%)	0 (0.0%)
**Reported AE by number of subjects**		
General disorders and administration site conditions	2 (15.4%)	1 (7.1%)
Influenza like illness	1 (7.7%)	1 (7.1%)
Pyrexia	1 (7.7%)	0 (0.0%)
Infections and infestations	0 (0.0%)	1 (7.1%)
Nasopharyngitis	0 (0.0%)	1 (7.1%)
Injury, poisoning and procedural complications	0 (0.0%)	2 (14.3%)
Foot fracture	0 (0.0%)	1 (7.1%)
Joint sprain	0 (0.0%)	1 (7.1%)
Investigations	2 (15.4%)	0 (0.0%)
Hepatic enzyme increased	2 (15.4%)	0 (0.0%)
Metabolism and nutrition disorders	0 (0.0%)	1 (7.1%)
Hypercholesterolaemia	0 (0.0%)	1 (7.1%)
Musculoskeletal and connective tissue disorders	1 (7.7%)	1 (7.1%)
Arthralgia	1 (7.7%)	0 (0.0%)
Bursitis	0 (0.0%)	1 (7.1%)
Nervous system disorders	6 (46.2%)	4 (28.6%)
Headache	2 (15.4%)	0 (0.0%)
Mononeuropathy	0 (0.0%)	1 (7.1%)
Multiple slcerosis relapse	6 (46.2%)	2 (14.3%)
Tension headache	0 (0.0%)	1 (7.1%)
Vascular disorders	0 (0.0%)	1 (7.1%)
Hypertension	0 (0.0%)	1 (7.1%)

AE: adverse event; N: number.

## Discussion

Atorvastatin 40 mg added to IFNB-1b did not have any beneficial effect on RRMS compared to IFNB-1b monotherapy over a period of 24 months. There were no significant differences in the primary or secondary endpoints between the two treatment groups. Non-siginificant trends were due to baseline differences and not treatment. The combination of atorvastatin and IFNB-1b was well tolerated and did not cause unexpected or severe side effects. All AEs were similar in both groups. Especially clinical relevant elevated liver enzymes did not occur more frequently in the atorvastatin/IFNB-1b group and unlike in the core study no dose adaptation of atorvastatin was necessary [8].

The results of the SWABIMS and SWABIMS Extension study are in line with several other combination trials of statins and IFNB in RRMS (Table 1) [8]–[15]. In this regard, there is actually no evidence to support the use of either atorvastatin or simvastatin as an adjunctive therapy to IFNB in RRMS [28], [29]. Adding atorvastatin or simvastatin to IFNB resulted to be safe and well tolerated without the occurance of serious side effects [28], [29].

In addition, SWABIMS and the SWABIMS extension study do not indicate a harmful effect of atorvastatin in addition to IFNB-1b on the course of RRMS as well. This, however, is still controversial due to individual data suggesting it [11], [14], [30], [31].

The safe and well tolerated use of statins in combination with IFNB in MS patients is of importance because MS patients with vascular risk factors and vascular disease have a more rapid disability progression than MS patients without [32], [33]. SWABIMS and the SWABIMS Extension Study in conjunction with other studies suggest that atorvastatin and simvastatin can be used for prevention of vascular events in MS patients who need a lipid-lowering therapy.

These conclusions on atorvastatin and simvastatin are likely to apply for other statins as well given the comparable immunomodulatory properties of the different statins in experimental studies [6], [34]. However, this has to be proven in separate clinical studies.

In contrast to the negative clinical studies, statins and IFNB have additive anti-inflammatory and immunomodulatory effects in vitro [34]. A possible explanation for this contradiction could be antagonistic effects of both drugs. Statins inhibit the STAT1 phosphorylation which is an important signaling pathway for IFNB, antagonize the inhibitory effect of IFNB on the proteolytic activity on MMP-2 and MMP-9, and reduce IFNB function and type 1 interferon responses in RRMS patients [34]–[37].

However, the question whether statins alone or in combination with other MS therapeutics could be beneficial in MS has not been studied and has yet to be answered. Statins have anti-inflammatory and immunomodulatory effects in experimetal studies including studies in “Experimental allergic encephalomyelitis” (EAE), the animal model of MS [3]–[6], [34]. Furthermore, an open-label, single-arm study evaluating simvastatin 80 mg/d in 30 RRMS patients showed a significant decrease in the number and volume of Gd-enhancing lesions [38]. A recent randomized, double-blind, placebo-controlled phase II trial, that is not published yet, showed a benificial effect of simvastatin 80 mg/d over two years in 140 secondary-progressive MS patients on disease progression (EDSS) and brain atrophy measures (brain boundary shift integrals) but no effect on relapse rate or T2 lesion activity [39]. These results indicate a possible positive effect of statins as monotherapy in MS. The latter study additionally emphasises a predominatly neuroprotective effect of statins in MS. This of course needs further investigation of statins in MS alone or in comination with other MS therapeutics.

There are limitations of the SWABIMS Extension Study. The number of patients was low due to the mentioned loss of patients caused by a safety analysis. Despite of the reduced statistical power, the results of the study still give meaningful informations with regard to the efficacy and safety of statins added to IFNB in the treatment of MS over a period of 24 month. Furthermore, it was not placebo-controlled because at the time of study planning and initiation an identical placebo was not available. We therefore chose a prospective randomized rater-blinded end-point study design. Nevertheless, the evaluating clinicians and neuroradiologists assessing MR endpoints were blinded. Another limitation might be the dose of atorvastatin. In vascular disease higher doses of atorvastatin are more effective than lower doses. However, the optimal immunomodulatory dosage is unknown and it is not certain that higher doses yield higher efficacy. Therefore and for safety reasons we chose a daily dose of 40 mg atorvastatin.

## Conclusions

In conclusion, atorvastatin 40 mg/d in addition to IFNB-1b did not have any beneficial effects on RRMS compared to IFNB-1b monotherapy over a period of 24 months. There is actually no evidence to support the use of atorvastatin 40 mg/d as an adjunctive therapy to IFNB in RRMS. The combination therapy was well tolerated and safe without the occurance of severe or unexpected side effects.

## Supporting Information

Checklist S1 **CONSORT Checklist.**
(DOC)

Protocol S1 **Trial Protocol.**
(DOC)
