# Supplementary material for: Atorvastatin Added to Interferon Beta for Relapsing Multiple Sclerosis: 12-Month Treatment Extension of the Randomized Multicenter SWABIMS Trial
Source: PLoS One. 2014 Jan 30;9(1):e86663. doi: 10.1371/journal.pone.0086663 (PMC3907426; doi:10.1371/journal.pone.0086663)
Supplement: Protocol S1 — Trial Protocol. (DOC) [file pone.0086663.s002.doc]

# Title page

Efficacy, safety and tolerability of Atorvastatin 40 mg in patients with relapsing-remitting multiple sclerosis treated with interferon-beta-1b.

**Multi-center, randomized, rater-blinded, parallel-group-study in Switzerland**

**SWiss Atorvastatin and Interferon-Beta 1b Trial In Multiple Sclerosis - Follow up Study**

**Protocol No. CWCNS012 “SWABIMS Follow up-study”**

Authors: Prof. Dr. med. H. Mattle Date: 21.03.2007

**Dr. med. Ch. Kamm**

**Confidential**

Clinical Study Phase: IIb

# Synopsis

**Title**  Efficacy, safety and tolerability of Atorvastatin 40 mg

in patients with relapsing-remitting multiple sclerosis treated with interferon-beta-1b - Follow up study

**SW**iss **A**torvastatin and Interferon-**B**eta 1b Trial **I**n **M**ultiple **S**clerosis **Follow up-study**

**Short title “SWABIMS Follow up-study”**

**Study phase**  Phase IIb study

**Study design** Multi-center, randomized, rater-blinded, parallel-group-study in Switzerland

**Investigational product, dosage,** Atorvastatin 40 mg every day (oral) plus Interferon-beta

**route of administration** 1b 250μg given subcutaneously (s.c.) every other day (e.o.d)

**Reference product, dosage,** Interferon-beta-1b 250μg given

**route of administration** subcutaneously (s.c.) every other day (e.o.d)

###### Indication Relapsing-remitting multiple sclerosis (RR-MS)

**Study objectives** Comparison of efficacy, safety and tolerability of combination of Atorvastatin 40 mg (per os) daily and Interferon-beta-1b e.o.d in patients with relapsing-remitting or secondary progressive forms of multiple sclerosis compared to monotherapy with Interferon-beta-1b e.o.d over a period of **12 months after successful finishing the SWABIMS-study**.

**Primary Endpoint** Efficacy of Atorvastatin as add-on therapy to a basic treatment with Interferon-beta-1b measured by the proportion of patients with new T2 lesions after 12 months of treatment.

**Secondary Endpoints** Gd-enhancing lesions on T1-weighted images after 12 months of treatment.

Total T2-hyperintense lesion volume (burden of disease, BOD) after 12 months of treatment.

Cortical atrophy (changes in brain volume, changes in grey matter and white matter) on MRI after 12 months of treatment

Clinical disease progression (EDSS, MSFC)

Functional systems scores (of EDSS and MSFC)

Number of relapse-free patients after 12 months of treatment

Relapse rate after 12 months of treatment

Time to first relapse

Sieric levels of anti-MOG- and anti-MBP-antibodies

Sieric levels of NAb

**Patient population** Men and women with relapsing-remitting forms of Multiple Sclerosis (according to McDonald’s criteria), **that have successful finished the SWABIMS-study**.

**Study design and Methodology** Multi-center, rater-blinded, parallel-group, two arm, randomized study.

**Safety** Physical examination, registration of adverse events and laboratory analysis will be performed at scheduled visits and, if adverse events are spontaneously reported, also at unscheduled visits.

**Duration of treatment**  12 months

**Number of study centers** 8

**Total number of patients** Approx. 60

**Adverse events** As interviewed or reported spontaneously by the patients or as noted on physical or laboratory examinations

**Planned start and end date** April 2007 – April 2009

**of recruitment**

**Manufacturer(s) of the** Schering AG: Interferon-beta-1b (Betaferon®)

**investigational /** Pfizer AG: Atorvastatin (Sortis®)

**reference product(s)**

**Principal Investigator** Prof. Dr. Heinrich Mattle

University Hospital Bern, Department of Neurology, Freiburgstrasse, 3010 Bern

**Steering Committee** Prof. Dr. Heinrich Mattle, Dr. Christian Kamm, Prof. Dr. Ludwig Kappos, Prof. Dr. Norbert Goebels, Prof. Dr. Barbara Tettenborn, Dr. Felix Müller, Prof. Dr. Martin Müller, Dr. Filippo Donati, Dr. Guido Schwegler

**Writing Committee** Dr. Christian. Kamm, Prof. Heinrich Mattle

**MRI Core lab**  Department of Neuroradiology, University Hospital Bern

**Monitoring/Database Mangement** PharmaPart AG, Bahnhofstrasse 20, P.O. Box 173,

CH-8800 Thalwil

**Sponsor** Department of Neurology, University Hospital Bern, Freiburgstr. 3010 Bern

**Other participating centers** Department of Neurology, University Hospital Basel

Department of Neuroimmunology, University Hospital Zürich

Department of Neurology, Kantonsspital St. Gallen

Department of Neurology, Spitalzentrum Biel

Department of Neurology, Kantonsspital Münsterlingen

Department of Neurology, Kantonsspital Luzern

Department of Neurology, Kantonsspital Aarau

# Table of contents

[1 Title page 1](#__RefHeading___Toc162228541)

[2 Synopsis 2](#__RefHeading___Toc162228542)

[3 Table of contents 5](#__RefHeading___Toc162228543)

[4 Investigators and study administrative structures 9](#__RefHeading___Toc162228544)

[4.1 Investigators 9](#__RefHeading___Toc162228545)

[4.2 Steering committee 9](#__RefHeading___Toc162228546)

[Investigators: 9](#__RefHeading___Toc162228547)

[4.3 Central laboratory, CRO and other institutions for study conduct 10](#__RefHeading___Toc162228548)

[5 Introduction 12](#__RefHeading___Toc162228549)

[6 Study Objectives 15](#__RefHeading___Toc162228550)

[6.1 Primary endpoint 15](#__RefHeading___Toc162228551)

[6.2 Secondary endpoints 15](#__RefHeading___Toc162228552)

[7 Study Design 16](#__RefHeading___Toc162228553)

[7.1 Selection of study population 16](#__RefHeading___Toc162228554)

[7.1.1 Inclusion criteria 16](#__RefHeading___Toc162228555)

[7.1.2 Exclusion criteria 16](#__RefHeading___Toc162228556)

[7.1.3 Removal of patients from the study 18](#__RefHeading___Toc162228557)

[7.2 Treatments 19](#__RefHeading___Toc162228558)

[7.2.1 Investigational products 20](#__RefHeading___Toc162228559)

[7.2.2 Treatment groups 20](#__RefHeading___Toc162228560)

[7.2.3 Method of assigning patients to treatment groups 21](#__RefHeading___Toc162228561)

[7.2.4 Blinding 21](#__RefHeading___Toc162228562)

[7.2.5 Prior and concomitant therapy 21](#__RefHeading___Toc162228563)

[7.2.6 Relapse 21](#__RefHeading___Toc162228564)

[7.2.7 Drug accountability 22](#__RefHeading___Toc162228565)

[8 Efficacy and Safety Variables 23](#__RefHeading___Toc162228566)

[8.1 Efficacy variables 23](#__RefHeading___Toc162228567)

[8.1.1 Primary efficacy variables 23](#__RefHeading___Toc162228568)

[8.1.2 Secondary efficacy variables 23](#__RefHeading___Toc162228569)

[8.2 Safety variables 23](#__RefHeading___Toc162228570)

[8.2.1 Safety and Tolerability definition 23](#__RefHeading___Toc162228571)

[8.2.2 Laboratory measurements 23](#__RefHeading___Toc162228572)

[8.3 Assessment of patient characteristics 24](#__RefHeading___Toc162228573)

[8.3.1 Demographic data 24](#__RefHeading___Toc162228574)

[8.3.2 Vital signs 24](#__RefHeading___Toc162228575)

[8.3.3 Neurological examination 24](#__RefHeading___Toc162228576)

[8.3.4 Medical and surgical history 24](#__RefHeading___Toc162228577)

[8.3.5 MS history: 25](#__RefHeading___Toc162228578)

[8.3.6 Medication history 25](#__RefHeading___Toc162228579)

[8.3.7 Physical examination 25](#__RefHeading___Toc162228580)

[8.3.8 ECG 25](#__RefHeading___Toc162228581)

[8.4 Magnetic resonance imaging (MRI) 25](#__RefHeading___Toc162228582)

[8.5 Adverse events 25](#__RefHeading___Toc162228583)

[8.5.1 Documentation 26](#__RefHeading___Toc162228584)

[8.5.2 Categories/Definition 26](#__RefHeading___Toc162228585)

[8.5.3 Intensity 27](#__RefHeading___Toc162228586)

[8.5.4 Drug relationship 27](#__RefHeading___Toc162228587)

[8.5.5 Serious adverse events 28](#__RefHeading___Toc162228588)

[8.5.6 Expected adverse reactions 29](#__RefHeading___Toc162228589)

[8.5.7 Unexpected adverse reactions 30](#__RefHeading___Toc162228590)

[8.6 Dose reduction of Atorvastatin or Interferon-beta-1b 30](#__RefHeading___Toc162228591)

[8.6.1 Creatine phosphokinase (CPK): 30](#__RefHeading___Toc162228592)

[8.6.2 Elevated Transaminases/Leucocytes-count: 31](#__RefHeading___Toc162228593)

[9 Study Schedule 33](#__RefHeading___Toc162228594)

[9.1 Flow chart 33](#__RefHeading___Toc162228595)

[10 Activities 34](#__RefHeading___Toc162228596)

[10.1 Baseline visit (Day 1 visit) 34](#__RefHeading___Toc162228597)

[10.1.1 Procedures at baseline: 34](#__RefHeading___Toc162228598)

[10.2 Treatment phase 34](#__RefHeading___Toc162228599)

[10.2.1 Month 6 visit (+/- 7 days) 34](#__RefHeading___Toc162228600)

[10.2.2 Months 12 Visit (+/- 7 days) 35](#__RefHeading___Toc162228601)

[10.2.3 Months 3 and 9 Visits (+/- 7 days) 35](#__RefHeading___Toc162228602)

[10.2.4 Unscheduled Visits 35](#__RefHeading___Toc162228603)

[11 STATISTICAL METHODS 36](#__RefHeading___Toc162228604)

[11.1 Statistical evaluation 36](#__RefHeading___Toc162228605)

[11.1.1 Methods of statistical analysis 36](#__RefHeading___Toc162228606)

[11.1.2 Evaluable subjects and analysis population 36](#__RefHeading___Toc162228607)

[11.1.3 Study endpoints 36](#__RefHeading___Toc162228608)

[11.1.4 Statistical analyses 36](#__RefHeading___Toc162228609)

[11.1.5 Demographic and Baseline Characteristics 37](#__RefHeading___Toc162228610)

[11.1.6 Safety Analyses 37](#__RefHeading___Toc162228611)

[12 ETHICS 39](#__RefHeading___Toc162228612)

[12.1 Ethical conduct of the study 39](#__RefHeading___Toc162228613)

[12.2 Written Patient information and informed consent form 39](#__RefHeading___Toc162228614)

[12.3 Confidentiality 39](#__RefHeading___Toc162228615)

[13 Reference list 40](#__RefHeading___Toc162228616)

[14 List of Abbreviations 43](#__RefHeading___Toc162228617)

[15 Appendix 45](#__RefHeading___Toc162228618)

[15.1 Magnetic Resonance Imaging 45](#__RefHeading___Toc162228619)

[15.1.1 Overview 45](#__RefHeading___Toc162228620)

[15.1.2 Scanning requirements 45](#__RefHeading___Toc162228621)

[15.1.3 Imaging 46](#__RefHeading___Toc162228622)

[Sequence parameters, T1 axial scout: 46](#__RefHeading___Toc162228623)

[Sequence parameters, T1 coronal scout: 46](#__RefHeading___Toc162228624)

[Sequence parameters, T1 sagittal scout: 47](#__RefHeading___Toc162228625)

[T1-weighted images to check correct positioning: 47](#__RefHeading___Toc162228626)

[T2-weighted images to check the correct positioning: 48](#__RefHeading___Toc162228627)

[Dual-echo images: 48](#__RefHeading___Toc162228628)

[T1-weighted post-gadolinium images: 49](#__RefHeading___Toc162228629)

[15.1.4 Electronic data 50](#__RefHeading___Toc162228630)

[15.1.5 Archiving 51](#__RefHeading___Toc162228631)

[15.1.6 MRI scan analysis 51](#__RefHeading___Toc162228632)

[15.1.7 MRI scan quality control 51](#__RefHeading___Toc162228633)

[15.2 Acceptable ranges for laboratory findings 51](#__RefHeading___Toc162228634)

[16 Patient information and consent 53](#__RefHeading___Toc162228635)

[16.1 Patient Information 53](#__RefHeading___Toc162228636)

[16.2 Patient consent 59](#__RefHeading___Toc162228637)

# Investigators and study administrative structures

## Investigators

For each study center, the following roles are to be identified:

- Treating physician
- Evaluating physician
- MRI investigator

In addition, further support staff may be identified.

## Steering committee

### Investigators:

Prof. Dr. Heinrich Mattle Department of Neurology

Dr. Christian Kamm University Hospital Bern

Freiburgstrasse

3010 Bern

Tel: 031-6323332

Fax: 031-6320321

Prof. Dr. Norbert Goebels Department of Clinical Neuroimmunology

University of Zuerich

Frauenklinikstrasse 26

CH-8091 Zürich

Tel: +41-44-255-5515

Fax:+41-44-255-9765

Prof. Dr. Ludwig Kappos Neurologische Universitätsklinik

Universitätsspital Basel

Petersgraben 4, 4031 Basel

Tel: 0612654151
Fax: 061 265 41 00

Prof. Dr. Barbara Tettenborn Klinik für Neurologie, Kantonsspital St. Gallen,9007,St.Gallen
Tel: 071 / 494 16 52
Fax: 071 / 494 28 95

Dr. Filippo Donati Neurologische Abteilung

Spitalzentrum Biel

Vogelsang 84

2502 Biel/Bienne

Tel: 032 / 3242424

Fax: 032 325 55 41

Dr. Guido Schwegler Neurologische Klinik,

Kantonsspital Aarau

5001 Aarau

Tel: 062 / 628386675

Fax: 062 838 66 74

Prof. Dr. Martin Müller Neurologische Klinik

Kantonsspital Luzern

Spitalstrasse

6000 Luzern

Tel: 041 205 11 11

Fax: 041 205 21 68

Dr. Felix Müller Neurologische Klinik

Kantonsspital Münsterlingen

8596 Münsterlingen

Tel: 071 686 1111

Fax: 071 686 2564

## Central laboratory, CRO and other institutions for study conduct

**CRO**

###### PharmaPart AG

Bahnhofstrasse 20

P.O. Box 173

CH-8800 Thalwil

Switzerland

Tel: +41-(0)44 7235959

Fax: +41-(0)44 7235960

www.pharmapart.com

**Central Laboratory**

**Viollier AG**
Spalenring 145 / 147
4002 Basel

Tel: +41 61 486 11 11
Fax: +41 61 486 15 18

# Introduction

**Multiple Sclerosis** (MS) is the most common neurological disorder causing disability in young adults affecting approximately 1 in 1000 persons in western countries (1). The clinical manifestations usually begin at the age of 20 to 40 years with a median age of onset of 28 years with acute episodes of neurological dysfunction, followed by periods of partial or complete remission and clinical stability in between relapses (2). This relapsing-remitting phase (RR-MS) of the disease is usually followed by progressive clinical disability (secondary progressive phase, SP-MS). Sometimes, relapses and partial remissions are superimposed on the chronic progression (3; 4). In a minority of patients, the disease progresses from the onset and does not remit (primary progressive, PP-MS).

Multiple sclerosis is considered to be a chronic inflammatory demyelinating autoimmune disease of the central nervous system, which involves T-cell-mediated immune reactions destructing myelin sheaths and axons (26). The prevention of autoimmunity requires mechanisms to actively silence or tolerize self reactive T cells in the periphery. For example, expansion of CD25+CD4+ T _regulatory_ cells, which constitute 5 - 10 % of peripheral CD4+ T cells in humans, prevents autoimmunity by suppressing the activation of self-reactive T cells (27). It has been observed that professional antigen presenting dendritic cells (DC) may prime those CD25+CD4+ T _regulatory_ cells to acquire suppressive properties (28). In MS this mechanism of tolerance may malfunction.

At present, there is no cure for multiple sclerosis and the management of MS-patients requires treatment with disease-modifying agents such as interferon-beta or immunsuppressants such as mitoxantrone, azathioprine or methotrexate. Acute relapses are usually treated with corticosteroids.

**Interferon-beta-1b** (Betaseron®, Betaferon®) is a non-glycosylated recombinant human interferon-beta approved for high-frequency, subcutaneous (sc) administration in the treatment of multiple sclerosis. In a randomised, double-blind trial in patients with relapsing-remitting MS (RRMS), interferon-beta-1b 250 micro g (8 million International Units [MIU]) every other day reduced the annual relapse rate and increased the proportion of relapse-free patients compared to placebo. It also reduced relapse severity, hospitalisations, and disease activity assessed by magnetic resonance imaging (MRI), and lengthened the time to a next relapse (5). The effect of interferon-beta-1b as seen on MRI in relapsing remitting multiple sclerosis was an approximately two-third reduction of disease activity (6). Beta-Interferon has been shown to exert multiple modulatory effects on immune cells, e.g. change of cytokine expression patterns, suppression of matrix metalloproteinase 9 (MMP) and others (30).

**Statins** are lipid-lowering drugs which inhibit the 3-hydroxy-3-methylglutaryl-coenzyme A (HMG-CoA-) reductase, which is the main regulatory enzyme of cholesterol biosynthesis. They lower cardiovascular-related morbidity and mortality and have a favourable safety profile being in clinical use for almost two decades (7). Side effects include hepatotoxicity (<3%) and myopathy (0.1-0.5%) (8). Rhabdomyolysis is the most severe form of muscle damage associated with statin use, although it is rare (9). The tolerability and the safety of Atorvastatin treatment, in line with other statins, are well documented by several trials, also at high doses, presenting a good risk-benefit curve (22, 23). In recent years many studies have demonstrated, that statins have anti-inflammatory and immunomodulatory properties in addition to their lipid-lowering effects. Therefore, statins seem to have therapeutic potential in immune-mediated disorders such as multiple sclerosis (10, 14, 29).

In ***experimental allergic encephalomyelitis (EAE)***, the animal model for the human demyelinating disease multiple sclerosis, oral statins attenuate the severity of disease progression by preventing or reversing chronic or relapsing paralysis. Statin-treated animals show delayed onset of the first clinical signs and milder clinical signs (11, 12, 24).

Lovastatin blocks the infiltration of mononuclear cells into the central nervous system (CNS), blocks the expression of extracellular adhesion molecule Leukocyte function antigen-1 (LFA-1) on leucocytes, inhibits the induction of proinflammatory cytokines and blocks the loss of myelin as a result of inflammatory disease in the CNS of EAE rats (12, 15).

Atorvastatin modulates the clinical course of murine experimental autoimmune encephalitis (EAE) in preventing or reversing chronic or relapsing paralysis as well. It induces signal transducer and activator of transcription (STAT) 6-phosphorylation and secretion of anti-inflammatory Th2-cytokines (IL-4, IL-5, IL 10), transforms growth factor (TGF)-beta and conversely inhibits STAT4-phosphorylation and secretion of inflammatory Th1-cytokines (IFN-γ, IL-2, IL-12, TNF-a) (11, 13, 14). Furthermore atorvastatin promotes the differentiation of Th0 cells into Th2-cells and reduces the CNS infiltration. It acts as direct inhibitor of induction of MHC class II expression by IFN-γ and thus as repressor of MHC-class II mediated T-cell activation. This effect is due to inhibition of the inducible promoter IV of the class II transactivator (CIITA) and is observed in several cell-types, including primary human endothelial cells and monocyte-macrophages (14). In addition adoptive transfer of atorvastatin-treated CD4+T cells prevents induction of EAE in recipient mice, which implies that statin-treatment induces a population of regulatory T cells which can suppress clinical autoimmune disease (11). Atorvastatin has pleiotropic immunomodulatory effects and may be beneficial by activating the pathway of tolerance.

Statins may also have neuroprotective properties. They upregulate endothelial nitric oxide synthase (eNOS) and inhibit inducible nitric oxide synthase (iNOS), effects which are both potentially neuroprotective. Moreover, eccito-toxicity caused by overstimulation of glutamate receptors is a major cause of neuronal death after a brain insult (18, 19, 20, 21, 24). Ex vivo, statins in combination with IFN beta-1b show synergistic effects in inhibiting the proliferation of stimulated peripheral blood mononuclear cells (PBMC) in a dose-dependent manner. Furthermore, statins reduced the expression of activation-induced adhesion molecules on T cells, reduces MMP-9 and downregulate chemokine receptors on both B and T cells (16).

In an open-label, single-arm study in ***patients with relapsing-remitting multiple sclerosis*** on use of oral simvastatin (80 mg), the mean number and volume of gadolinium-enhancing lesions compared to pretreatment brain MRI scans declined by 44% and 41% respectively, while treatment was well tolerated (17). In a very small study lovastatin therapy showed beneficial effects on the expanded disability status scale (EDSS), relapse rate and Gd-enhancing lesions (25).

**Multiple sclerosis patients** respond variably to monotherapies, which could be explained by different subtypes of multiple sclerosis and multiple environmental and genetic factors. Oral statins decrease neuroinflammatory activity and clinical signs in experimental allergic encephalomyelitis and showed beneficial effect in smaller studies in multiple sclerosis patients. Therefore, statins may have favourable clinical effects in patients with relapsing-remitting disease as single therapy, or administrated as combination therapy with interferon beta. To determine the therapeutic effect of statins in multiple sclerosis, further investigations of statins as therapeutic agents for MS should be addressed in larger controlled clinical trials and longer observation periods.

In this study the effect of Atorvastatin as add-on therapy in patients with RR-MS, already treated with interferon-beta-1b vs. treatment with Interferon-beta-1b alone will be evaluated over another 12 months after finishing the SWABIMS Study as a Follow up study of the SWABIMS-Study. The treatment of each patient will be the same. Furthermore important safety and tolerability data will be generated.

# Study Objectives

The objectives of this study are to assess the efficacy, safety and tolerability of the combination of Atorvastatin 40mg p.o. daily and Interferon-beta-1b sc e.o.d compared to monotherapy with Interferon-beta-1b sc e.o.d in patients with relapsing-remitting multiple sclerosis **for another 12 months after successful finishing the SWABIMS-Study**.

## Primary endpoint

The primary endpoint of the study is to determine the efficacy of Atorvastatin as add-on therapy to a basic treatment with Interferon-beta-1b, evaluating the disease activity measured by the proportion of patients with new T2 lesions after 12 months of treatment.

## Secondary endpoints

- Gd-enhancing lesion on T1-weighted images after 12 months of treatment
- Change of total T2 hyperintense lesion volume after 12 months of treatment (burden of disease, BOD)
- Clinical disease progression (EDSS, MSFC)
- Functional system scores (of EDSS and MSFC)
- Number of relapse-free patients after 12 months of treatment
- Relapse rate after 12 months of treatment
- Time to first relapse
- Cortical atrophy (changes in brain volume, changes in grey matter and white matter) after 12 months of treatment
- Sieric levels of antibodies against myelin oligodendrocyte glycoprotein (anti-MOG) and against myelin basic protein (anti-MBP)
- Sierial levels of neutralizing Antibodies (Nab)

# Study Design

- Multi-center, rater-blinded, parallel-group, two arms, randomized study. Patients with relapsing-remitting forms of MS, **who have successfully finished the SWABIMS-Study**, respecting all inclusion/exclusion criteria, will be treated with the same medication they have received during the SWABIMS-Study for another 12 months directly after finishing the **SWABIMS-Study**.
- In the **SWABIMS-study**, 80 patients with relapsing-remitting MS were randomized to either an Interferon-beta-1b-monotherapy (subcutaneous every other day) **or** to a treatment with Interferon-beta-1b (subcutaneous every other day) plus Atorvastatin 40mg/d for 12 months after they were treated with Interferon-beta-1b (subcutaneous every other day) for 3 months.
- Enrolment of approx. 60 patients (one half in the Atorvastatin group) is planned.
- Patients providing written informed consent will be ***treated for 12 months***.

## Selection of study population

### Inclusion criteria

- **Patients, who have successfully finished the SWABIMS-Study.**
- Patients with relapsing-remitting forms of multiple sclerosis (according to McDonald’s criteria)
- Patients must agree to avoid macrolids, including erythromycin and clarithromycin for the whole study duration
- Availability and willingness to continue study visits and procedures through month 12, even if discontinuation of study drug is necessary
- A woman of child-bearing potential must agree to avoid pregnancy with appropriate means while on study. Hormonal contraceptives used for more than 3 months are acceptable if therapy remains constant throughout the study
- Written informed consent
- Adequate bone marrow, renal, and hepatic function as defined in appendix.

### Exclusion criteria

- Patients, who didn’t successfully finish the SWABIMS Study.
- Any disease other than multiple sclerosis that would better explain the patient’s signs and symptoms
- Primary progressive MS
- Uncontrolled, clinically significant heart diseases, such as arrhythmias, angina, or uncompensated congestive heart failure.
- Uncontrolled seizure disorder
- Myopathy or clinically significant liver disease
- Medical or psychiatric conditions that compromise the ability to give informed consent, to comply with the protocol, or to complete the study
- Inability, in the opinion of the principal investigator or staff, to comply with protocol requirements for the duration of the study
- Known hypersensitivity to either Atorvastatin or Interferon-beta or other human proteins including albumin
- Known hypersensitivity to gadolinium
- Inability to undergo a MRI scan
- A history of drug abuse in the 6 months prior to enter the study
- Patients will not be permitted to take any antilipidemic medications (refer to list below). Most other medications taken for treatment of chronic conditions is permitted, as well as most medications to treat minor treatment-emergent illness(es). Given the importance of Atorvastatin metabolism by cytochrome P450 3A4, only concomitant medications permitted in the package insert will be allowed during study participation. Consistent with the package insert, the following medications are expressly prohibited throughout the study:
- All lipid-lowering medications, including niacin, fibrates (e.g., clofibrate, fenofibrate, bezafibrate, gemfibrozil), bile acid sequestrants (e.g., cholestyramine, colestipol), and HMG-CoA reductase inhibitors (e.g. fluvastatin, pravastatin, simvastatin)
- Orlistat
- Macrolide antibiotics (e.g., erythromycin, azithromycin, clarithromycin)
- Azole antifungals (e.g., fluconazole, clotrimazole, itraconazole)
- Immunosuppressive medications (e.g., cyclosporine)
- Protease inhibitors
- Nefazodone
- Fish oils
- Grapefruit juice
- Previous (before start of study) therapy with any of the following: Monoclonal antibodies, mitoxantrone, cytotoxic or immunosuppressive therapy (excluding systemic steroids and adrenocoticotropic hormone; ACTH), total lymphoid irradiation.
- Participation in any other studies (**except the SWABIMS-Study**) involving investigational or marketed products, concomitantly or within 30 days prior to entry in the study
- Treatment with drugs which might interfere with the evaluation of study drugs during the study protocol
- Likelihood of requiring treatment during the study period with drugs not permitted by the study protocol

### Removal of patients from the study

- Every patient or his/her legal representative has the right to refuse further participation in the study at any time and without providing reasons. A patient's participation is to terminate immediately upon his/her request. The investigator should seek to obtain the reason.
- The patient may be withdrawn from the study at any time at the investigator’s discretion; the reason should be documented.
- Patients who withdraw from the study will not be replaced.
- There are two different types of patient **withdrawals** that may be decided:
  1. Withdrawal from further **study participation**; *i.e*. study treatment will be discontinued and no further data will be collected for the patient withdrawn. For this type of withdrawal, before withdrawal all efforts should be made to perform all assessments scheduled for the end-of-study visit (except MRI).
  2. Withdrawal from further **study medication** without termination of study participation.

If premature termination of study medication is decided for a patient, all efforts should be made to keep that patient on the original visit schedule until the regular end-of-study visit *(i.e.*, irrespective of study medication, each randomized patient should be followed up for the originally scheduled duration).

For both types of withdrawal, the following general rules apply: as far as possible, any withdrawal of a patient should be decided only after consultation with the sponsor.

The reasons for any withdrawal are to be fully documented on the case report form (CRF).

- **Withdrawal criteria**

A patient must be withdrawn from further **study participation** under the following circumstances:

1. Withdrawal of consent: every patient has the right to refuse further participation in the study at any time and without providing reasons.
2. Participation in any other clinical trial during the duration of this study.

A patient must be withdrawn from further **study medication** under the following circumstances:

1. Non-attendance of visits

Study medication must be withdrawn if a patient does not attend two consecutive scheduled visits without a major reason agreed on by the sponsor.

1. Unauthorized use of study drug.
2. Pregnancy
   Patients who have been withdrawn from study drug treatment because of pregnancy should not undergo MRI scans while pregnant.
3. Psychiatric conditions

In the case of severe depression, suicidal ideation, or attempted suicide, study medication must be interrupted until the patient’s psychiatric condition has been fully evaluated. A psychiatrist should be consulted whenever necessary. Treatment may be resumed only after consultation with the sponsor.

A patient may be withdrawn from further **study medication** under the following circumstances:

1. Laboratory abnormality or serious/severe adverse event if dose modifications in case of toxicities don’t counteract the abnormality.
2. At the discretion of the treating physician.

The development of conditions which would have prevented a patient’s entry into the study according to the selection criteria is no reason *per se* for withdrawal. However, the withdrawal in such cases remains at the discretion of the treating physician; in these cases, consultation with the sponsor before such a decision is requested.

## Treatments

The investigator should maintain adequate records of the receipt and disposition of all study drugs supplied. Any unused medication will be returned to the sponsor at the end of the study. The investigator shall be responsible for ensuring that the study medication is stored in a cool, secure, limited access area, protected from extremes of light, temperature, and humidity.

All drug supplies, including any commercially available background and/or comparator drugs, must be stored in accordance with the manufacturers’ instructions. Study drug supplies must be stored separately from normal hospital/practice stocks. Until dispensed to the subjects, the study medication will be stored in a securely locked area, only accessible to authorized personnel.

### Investigational products

#### Interferon-beta-1b (Betaferon®, Betaseron®, Schering)

Interferon-beta-1b (Betaseron®, Betaferon®) is a non-glycosylated recombinant human interferon-beta approved for high-frequency, subcutaneous (sc) administration in the treatment of multiple sclerosis. In a randomised, double-blind trial in patients with relapsing-remitting MS (RRMS), interferon-beta-1b 250 micro g (8 million International Units [MIU]) every other day reduced the annual relapse rate and increased the proportion of relapse-free patients compared to placebo. It also reduced relapse severity, hospitalisations, and disease activity assessed by magnetic resonance imaging (MRI), and lengthened the time to a first relapse (5). The effect of interferon-beta-1b as seen on MRI in relapsing remitting multiple sclerosis was an approximately two-third reduction of disease activity.

#### Atorvastatin (Sortis®, Pfizer)

**Chemical name:** Atorvastatin calcium is [R-(R^*^, R^*^)]-2-(4-fluorophenyl)-β,δ-dihidroxy-5-(1-methylethyl)-3-phenyl-4-[(phenylamino)carbonyl]-1H-pyrrole-1-heptanoic acid, calcium salt (2:1)

**Dosage form:** oral tablets

**Strength:** 40 mg

Atorvastatin is a selective, competitive inhibitor of HMG-CoA reductase, the rate-limiting inhibitor enzyme that converts 3-hydroxy-3-methylglutaryl-coenzyme A to mevalonate, a precursor of steroids, including cholesterol. Cholesterol and triglycerides circulate in the bloodstream as party of lipoprotein complexes. With ultracentrifugation, these complexes separate into HDL (high-density lipoprotein), IDL (intermediate-density lipoprotein), LDL (low-density lipoprotein), and VLDL (very-low-density lipoprotein) fractions. Triglycerides (TG) and cholesterol in the liver are incorporated into VLDL and released into the plasma for delivery to peripheral tissues. LDL is formed from VLDL and is catabolized primarily through the high-affinity LDL receptor.

### Treatment groups

#### Group 1 (Atorvastatin)

SWABIMS Study: After a treatment period of three months with Interferon-beta-1b 250 micro g (8 million International Units [MIU]) subcutaneously every other day, oral Atorvastatin (40 mg/d) was administered in addition for 12 months.

**SWABIMS Follow up Study:** The treatment with Interferon-beta-1b (subcutaneous every other day) and oral Atorvastatin (40mg/d) will be continued immediately after the end of the SWABIMS Study for 12 month.

#### Group 2 (No Atorvastatin)

SWABIMS Study: Interferon-beta-1b 250 micro g (8 million International Units [MIU]) was administered subcutaneously every other day as indicated by the manufacturers’ instructions for 15 months.

**SWABIMS Follow up Study:** The treatment with Interferon-beta-1b (subcutaneous every other day) will be continued for 12 months.

### Method of assigning patients to treatment groups

The treatment groups will be the same as in the SWABIMS-Study. Each patient continues the medication he/she received in the SWABIMS Study.

### Blinding

This is a rater-blinded study. All patients and the treating physician will be aware whether Atorvastatin is added to the Interferon-beta-1b therapy or not.

Treatment will be administered by the “treating physician” (TP). He/she will acknowledge the treatment administered, he/she will be responsible for patient eligibility, for treatment of adverse events, and for safety examinations.

”Examining physicians” (EP) will be responsible for disability scoring (EDSS, MSFC). They will not be informed whether patients are receiving Atorvastatin or not. Results of scoring will be recorded in a separate sheet. The EP will not acknowledge the treatment groups. Safety issues and other questions should not be discussed between TP and EP. Patients and TP should be informed not to discuss any safety questions with the EP.

In addition, the MRI evaluations will be performed with readers blinded to the treatment group.

Unblinding of the evaluating physician or his/her staff should generally be avoided. Single case unblinding is allowed when the actual treatment must be revealed in order to influence urgent medical care.

### Prior and concomitant therapy

Concomitant therapy with NSAID’s or acetaminophen will be at the investigator’s discretion. During the study if a patient experiences an exacerbation of symptoms related to his/her underlying MS, treatment can be given at the discretion of the treating physician. Use of medications to treat conditions unrelated to MS is allowed and will be administered at the discretion of the investigator.

### Relapse

#### Relapse definition/assessment

**All** of the following criteria (1. to 3.) have to be met for a relapse to be established:

1. neurological abnormality, either newly appearing or re-appearing, with abnormality specified by both
   1. neurological abnormality separated by at least 30 days from onset of a preceding clinical event
   2. neurological abnormality lasting for > 24 hours
2. Absence of fever or known infection (fever = > 37.5°C)
3. objective neurological impairment, correlating with the patient`s reported symptoms
   1. increase in at least one of the functional systems of the EDSS score
   2. increase of the total EDSS score

The occurrence of fatigue, mental symptoms, and/or vegetative symptoms without any additional symptom will not be classified as a relapse.

#### Relapse treatment

We recommend to treat relapses during the study with corticosteroids in form of intravenous methylprednisolone at a dose of 500mg daily for 5 consecutive days i.v. with or without tapering-out. The decision to treat an exacerbation is at the discretion of the treating physician.

### Drug accountability

Drug accountability will be maintained at the research site.

The investigator will keep a record of the investigational product (Atorvastatin) dispensed. The investigational product must be stored in a locked storage facility and protected from unauthorized access.

Patients must return used or unused investigational product in the original packages upon termination of the study at the latest.

# Efficacy and Safety Variables

## Efficacy variables

The objectives of this study are to assess the efficacy, safety and tolerability of the combination of Atorvastatin 40 mg p.o. daily and Interferon-beta-1b sc e.o.d compared to monotherapy with Interferon-beta-1b sc e.o.d in patients with multiple sclerosis.

### Primary efficacy variables

The primary endpoint of the study is to determine the efficacy of Atorvastatin as add-on therapy to a basic treatment with Interferon-beta-1b evaluating the disease activity measured by the proportion of patients with new T2 lesions after 12 months of treatment.

### Secondary efficacy variables

The following points will be analyzed comparing group one and two.

- Gd-enhancing lesion on T1-weighted images after 12 months of treatment.
- Change of total T2 hyperintense lesion volume (burden of disease, BOD)
- Clinical disease progression (EDSS, MSFC)
- Functional systems subscores (of EDSS and MSFC)
- Number of patients relapse-free after 12 months of treatment
- Relapse rate after 12 months of treatment
- Cortical atrophy (changes in brain volume, changes in grey matter and white matter) after 12 months of treatment
- Sieric levels of anti-MOG- and anti-MBP-antibodies
- Sierial levels of neutralizing Antibodies (Nab)

## Safety variables

### Safety and Tolerability definition

Safety and tolerability will be measured by reporting of adverse events, safety laboratory measurements and drop out rate due to adverse events.

### Laboratory measurements

#### Serum Chemistry

Sodium, Potassium, Chloride, Calcium, Urea-N, Creatinine, ASAT (SGOT), ALAT (SGPT), GGT, alkaline phospatase, Uric Acid, total and direct bilirubin, total protein and albumin, Glucose

#### Serum lipid profile

Triglycerides, total cholesterol, free cholesterol, HDL, LDL

#### Hematology

RBC, WBC, Differential count, Platelet Count, Hemoglobin (Hb), Hematocrit, absolute lymphocyte count

#### Pregnancy Test in Women

Serum beta-HCG

#### Creatine phosphokinase (CPK)

Total CPK, isoenzymes only if total CPK is more than twice the normal upper range.

#### Immunology:

Neutralizing antibodies (Nab), anti-MOG- and anti-MBP-Antibodies

#### Thyroid Profile

TSH

## Assessment of patient characteristics

### Demographic data

Documentation of date of birth, sex, and ethnic group

### Vital signs

Height, weight, temperature, heart rate (for 1 min, after being seated for 3 min), and systolic and diastolic blood pressure (after being seated for 3 min) will be measured. In case of clinically significant deviations, the measurements will be repeated before documentation.

### Neurological examination

Will be performed by the Evaluating Physician. The results of the standardized neurological examination and Kurtzke EDSS score and MFSC will be recorded.

### Medical and surgical history

The treating physician will ask about relevant diseases of the following organs/systems: skin, eyes, ears, nose, and throat, head and neck (including the thyroid), lungs, heart, breasts, liver, gastro-intestinal system, lymph nodes, musculoskeletal system (including extremities and spine), genito-urinary system. History of allergies will be queried, as well as other abnormalities (if indicated). Furthermore, patients will also be asked if they have a history of alcohol or drug abuse, and a history of psychiatric disorder, with particular regard to known depression or suicidal tendencies. Any previous disease or surgery reported will also be documented.

The contraceptive method used will be recorded, and if applicable, the age at menopause. Patients of childbearing potential will be instructed to discontinue study medication if they become pregnant and immediately notify the Treating Physician.

### MS history:

Date of onset and diagnosis of MS, symptoms, recovery, number of relapses during the past two years

### Medication history

The generic names of the preparations should be recorded and for compound preparations, the trade name should be recorded. For each medication, the indication, dose, unit, frequency, route of administration, and dates of first and last administration will be documented.

### Physical examination

The physical examination will be performed by the Treating Physician. Any abnormal physical examination findings or ongoing symptoms or diseases (except neurological findings related to multiple sclerosis) observed are to be reported. The date when the symptoms first occurred, and the stop date, if appropriate, will be documented.

### ECG

A 12-lead ECG will be performed.

## Magnetic resonance imaging (MRI)

All patients will undergo MRI examination at the time of study entry and at month 12. The study scans should be performed within 14 days of the scheduled visit. If a patient receives a high dose of corticosteroids to treat an exacerbation, then the MRI scan will be postponed until 30 days after the treatment is completed. MRI scans are to be performed following standardized MRI guidelines (see detailed protocol) and will include pre and post contrast,T1 images and T2 images. The MRI scans will be analyzed centrally in a blinded fashion at the MRI core lab. MRI is used in this study as a parameter of activity. Recent studies have shown that the number of T2 positive areas and the number of new lesions on the T2-weighted images considered correlates with the activity detected by MRI with gadolinium, and shows a positive trend in the progression of the disease. Furthermore, new T2 lesions reflect disease activity over the whole interval of 3 months, whereas gadolinium-enhancing lesions reflect only the previous month. A highly concentrated contrast agent will be used for the T1 sequences, to evaluate disease activity of the last month by means of gadolinium-enhancing lesions. T1-weighted sequences of MRI scans will be performed with high concentrated gadobutrol. The contrast medium may occasionally cause nausea and vomiting, it may cause warmth and pain at the injection site. Allergic reactions occur very rarely (anaphylactic shock in 13 of 5 million applications, based on post-marketing surveillance) and in extremely rare instances can be potentially serious.

## Adverse events

An **AE** is defined as any untoward medical occurrence in a patient or clinical investigation subject (volunteer) administered an investigational medicinal product and which does not necessarily have a causal relationship with this treatment. An AE can therefore be any unfavorable and unintended sign (including an abnormal but clinical significant laboratory finding), symptom, or disease temporally associated with the use of a medicinal (investigational) product, whether or not the AE is considered related to the medicinal (investigational) product. AEs are to be coded using an internationally recognized dictionary.

### Documentation

The patient should be closely observed by the investigator. In case of drug-related AE (i.e.,‘possible’, ‘probable’ or ‘definite’ drug relationship assessment), the investigator is urged to monitor the patient and document the outcome on a separate form (additional CRF page)*.*

Any AEs (observed, volunteered, or elicited) are to be documented in detail indicated on the CRF.

The following information is required:

- The **date of onset** of any AEs
- The fact whether an AE is considered to be serious (**SAE** no/yes)
- The maximum **intensity** (mild, moderate or severe; for definitions, see below)
- The main **pattern** (for definitions, see below)
- **Study drug action** because of this AE (drug withdrawn, dose not changed, etc.)
- **Details** of AE-related study drug actions (free text), if applicable
- Specific **treatment** of the AE (no/yes)
- The **study drug relationship** of the AE to the investigational product (for definitions, see below)
- The **outcome** of the adverse event (recovered, recovering, not recovered, recovered with residual effects, fatal, unknown)
- **Date ended** (if recovered)
- An assessment of the **seriousness** of the event will be made by the investigator, who is to complete a special form provided by the sponsor in the case of a serious adverse event (SAE). However, SAEs will also be recorded briefly on the "Adverse Event Form" of the CRF. A definition of serious adverse events is provided below.

### Categories/Definition

**Every study drug administration**:

Events that occur in a clear time relationship to every study drug administration

**Intermittent:**

Regular or irregular repeating events that are clearly of the same kind and same cause, but not clearly time related to study drug administration

**Continuous**:

Events that are continuously present within the whole time period which is covered by the form, but not clearly time related to study drug administration

**Other**:

All other pattern, need to be specified in the following text field

### Intensity

The investigator is to classify the intensity of an **AE** according to the following definitions:

**Mild**: The intensity of the event is assessed as mild, taking into account the possible range of the intensity of the event.

**Moderate:** The intensity of the event is assessed as moderate, taking into account the possible range of the intensity of the event.

**Severe:** The intensity of the event is assessed as severe, taking into account the possible range of the intensity of the event.

### Drug relationship

The investigator has to classify the drug relationship of an AE according to the following definitions:

| **Categories** | Definition |
| --- | --- |
| None | The time course between administration of the study drug and occurrence or worsening of the AE is out a causal relationship and/or another cause is confirmed and no indication of involvement of the study drug in the occurrence/worsening of the AE exists. |
| **Unlikely** | The time course between administration of the study drug and occurrence or worsening of the AE makes a causal relationship unlikely and/or the known effects of the study drug or of the substance class provide no indication of involvement in occurrence/worsening of the AE and another cause adequately explaining the AE is known and/or regarding the occurrence/worsening of the AE a plausible causal chain may be deduced from the known effects of the study drug or the substance class, but another cause is much more probable and/or another cause is confirmed and involvement of the study drug in the occurrence/worsening of the adverse event is unlikely. |
| **Possible** | Regarding the occurrence/worsening of the AE, a plausible causal chain may be deduced from the pharmacological properties of the study drug or the substance class, but another cause just as likely to be involved is also known or although the pharmacological properties of the study drug or the substance class provide no indication of involvement in the occurrence/worsening of the AE, no other cause gives adequate explanation |
| **Probable** | The pharmacological properties of the study drug or of the substance class and/or the course of the AE after dechallenge and, if applicable, after rechallenge and/or specific tests (e.g. positive allergy test, antibodies against study drug/metabolites) suggest involvement of the study drug in the occurrence/worsening of the AE, although another cause cannot be ruled out. |
| **Definite** | The pharmacological properties of the study drug or of the substance class and the course of the AE after dechallenge and, if applicable, after rechallenge and specific tests (e.g. positive allergy test, antibodies against study drug/metabolites) indicate involvement of the study drug in the occurrence/worsening of the AE and no indication of other causes exists. |

### Serious adverse events

The following SAE definition is based on ICH guidelines and the final rule issued by the Food and Drug Administration (FDA) and effective 6 Apr 1998.

A **SAE** is classified as any untoward medical occurrence that at any dose

- results in death, or
- is life-threatening, or
- requires inpatient hospitalization (except pre-planned hospitalization and **hospitalisation due to relapse or other medical problems of multiple sclerosis**) or prolongation of existing hospitalization, or
- results in persistent or significant disability/incapacity, or
- involves malignancy or is a congenital anomaly / birth defect

Medical and scientific judgment should be exercised in deciding whether expedited reporting is appropriate in other situations, such as important medical events that may not be immediately life-threatening or result in death or hospitalization but may jeopardize the volunteer or may require intervention to prevent one of the other outcomes listed in the definition above. These should also usually be considered serious. Examples of such events are intensive treatment in an emergency room or at home for allergic bronchospasm; blood dyscrasias or convulsions that do not result in hospitalization; or development of drug dependency or drug abuse.

The investigator should take appropriate diagnostic and therapeutic measures to minimize the risk to the patient. Where appropriate he / she should take diagnostic measures to collect evidence for clarification of the relationship between the SAE and the investigational product.

The investigator must submit a complete SAE report for all SAEs, regardless of a possible causal relationship, to the primary contact person at the **CRO** **immediately** (at the latest within 24 hours of having gained knowledge of the event).

**Address:**

**PharmaPart AG**

**Bahnhofstrasse 20**

**P.O. Box 173**

**CH-8800 Thalwil**

**Switzerland**

**Tel: +41-(0)44 723 59 59**

**Fax: +41-(0)44 723 59 60**

**www.pharmapart.com**

This report is to be sent per Fax on the SAE Report Form provided by the CRO. The CRO immediately will notify the Study Manager and the Study Coordinating CRA. The investigator is required to document in full the course of the SAE and any therapy given, including any relevant findings / records in the report. The investigator will also inform the CRO of the relevant follow up information and the outcome of the SAE as soon as possible using the standard form.

For reported deaths, the investigator is required to supply the sponsor with any additional available information (e.g.: autopsy reports and terminal medical reports).

All AEs and SAEs after enrollment of the subject (as soon as the informed consent has been signed) must be documented.

### Expected adverse reactions

**Atorvastatin** is generally well-tolerated and side effects are rare. Minor side effects include constipation, [diarrhea](http://www.medicinenet.com/script/main/art.asp?ArticleKey=1900), fatigue, gas, pruritus, heartburn, and headache. Major side effects include [abdominal pain](http://www.medicinenet.com/script/main/art.asp?ArticleKey=1908) or cramps, blurred vision, [dizziness](http://www.medicinenet.com/script/main/art.asp?ArticleKey=8004), easy bruising or bleeding, itching, muscle pain or cramps, rash, and yellowing of the skin or eyes. [An](javascript:defwindow('An')) [apparent](javascript:defwindow('apparent')) hypersensitivity [syndrome](javascript:defwindow('syndrome')) has been reported rarely. Atorvastatin, like other inhibitors of HMG-CoA reductase, occasionally causes [myopathy](javascript:defwindow('myopathy')) manifested as [muscle](javascript:defwindow('muscle')) pain, [tenderness](javascript:defwindow('tenderness')) or [weakness](javascript:defwindow('weakness')). Myopathy sometimes takes the [form](javascript:defwindow('form')) of [rhabdomyolysis](javascript:defwindow('rhabdomyolysis')) with or without [acute](javascript:defwindow('acute')) [renal](javascript:defwindow('renal')) [failure](javascript:defwindow('failure')) [secondary](javascript:defwindow('secondary')) to myoglobinuria. The [risk](javascript:defwindow('risk')) of [myopathy](javascript:defwindow('myopathy')) is increased by high levels of HMG-CoA [reductase](javascript:defwindow('reductase')) [inhibitory](javascript:defwindow('inhibitory')) [activity](javascript:defwindow('activity')) in plasma. Laboratory abnormalities may include elevated bilirubin, alkaline phosphatase, [thyroid](javascript:defwindow('thyroid')) function abnormalities.

Marked persistent increases of [serum](javascript:defwindow('serum')) transaminases have been noted. About 5% of patients had elevations of CK levels of 3 or more times the normal [value](javascript:defwindow('value')) on one or more occasions. This was attributable to the noncardiac fraction of CK. Muscle [pain](javascript:defwindow('pain')) or [dysfunction](javascript:defwindow('dysfunction')) usually was not reported.

Recenty, data from a retrospective analysis (conducted using pooled data from 44 completed trials comprising 16,495 dyslipidemic patients treated with atorvastatin, placebo, and other statins) has been reported. Only 3% of atorvastatin-treated patients withdrew from studies due to treatment-associated adverse events, compared with 1% of those on placebo and 4% of those receiving other statins; the most frequently reported treatment-associated adverse events were related to the digestive system. Serious adverse events were rare and seldom led to withdrawal. Persistent elevations in hepatic transaminases to >3 times the upper limit of normal (ULN) were experienced by 0.5% of atorvastatin-treated patients. A persistent elevation in creatine phosphokinase (CPK) was observed in only 1 atorvastatin-treated patient and was not associated with myopathy. No cases of rhabdomyolysis or myopathy were reported.

**Interferon-beta-1b** 250 micro g (8 million International Units [MIU]) administered subcutaneously every other day can cause injection side reactions such as erythema, pain or swelling or “flu-like” symptoms such as fever, myalgia, chills, headache or arthralgia. Elevation of hepatic enzymes and lowered WBC-counts were also reported.

### Unexpected adverse reactions

Unexpected adverse drug reaction is defined as an adverse reaction whose nature and severity is not consistent with the applicable product information.

## Dose reduction of Atorvastatin or Interferon-beta-1b

If an adverse event occurs due to Atorvastatin or Interferon beta-1b administration, haematological, serum chemistry and CPK will be tested.

### Creatine phosphokinase (CPK):

- CPK elevated factor 3:
  - CPK-control in two weeks
- CPK elevated factor 5:
- Atorvastatin will be reduced from 40mg to 20mg, if the isoenzyme CPK-MB is normal and in the absence of clinical symptoms (e.g. muscle pain, cramps)
- CPK elevated factor 10:
- Atorvastatin will be stopped

After two weeks haematological, serum chemistry and CPK testing will be repeated, and in the absence of normalized values, the Atorvastatin dose will be reduced/stopped until normalization of values is reached.

### Elevated Transaminases/Leucocytes-count:

#### Patients with interferon-beta-1b monotherapy

1. leucocyte-count < 2500:
   - reduce dosage of interferon by 1/2 for 2 weeks
2. leucocyte-count < 2000:
   - stop interferon for 2 weeks
3. elevated tramsaminases times 3 of the upper value
   - reduce dosage of interferon by 1/2 for 2 weeks
4. elevated tramsaminases times 5 of the upper value
   - stop interferon for 2 weeks

After normalisation the therapy can be adapted

1. in case 1 and 3, the original dose can be administered
2. in case 2 and 4, interferon-beta-1b be must be slowly elevated

- ¼ of the final dosage for 1 week
- ½ of the final dosage for 1 week
- ¾ of the final dosage for 1 week
- full dose after 4 weeks

#### Patients with Interferon-beta-1b and Atorvastatin-therapy

- An elevation of the transaminases 3x the ULN can be tolerated.
- In case of an elevation of the transaminases the dose of Atorvastatin will be reduced first.

1. elevated tramsaminases ***times 3*** the ULN
   - halve the dosage of Atorvastatin (= 20 mg/d) for 2 weeks
2. elevated tramsaminases ***times 5*** the ULN
   - stop Atovastatin for 2 weeks

After normalisation the therapy can be adapted

- - in case 1 the original dose can be administered
  - in case 2 Atorvastatin will be administered 20 mg/d for 2 weeks and afterwards 40 mg/d

while increasing the dose, laboratory controls of the elevated values should be done every two weeks.

# Study Schedule

## Flow chart

The Table 1 provides a detailed schedule of activities and evaluations to be performed throughout the study.

**Table 1. Schedule of Activities and Evaluations**

| Activities and assessments | Baseline Day 1 | Month 3  🕻 telephone | Month 6 (+/- 7 days) | Month 9  🕻 telephone | Month 12 (+/- 7 days) |
| --- | --- | --- | --- | --- | --- |
| Written informed consent | ● |  |  |  |  |
| In-/exclusion criteria | ● |  |  |  |  |
| Demographic data | ● |  |  |  |  |
| MS history | ● |  |  |  |  |
| Medical/surgical history | ● |  |  |  |  |
| Physical examination | ● |  | ● |  | ● |
| Medication history | ● |  |  |  |  |
| Lab evaluation | 1,2 |  | 1 |  | 1,2 |
| Pregnancy test | ● |  |  |  |  |
| Urinalysis | ● |  |  |  | ● |
| Vital signs | ● |  | ● |  | ● |
| ECG | ● |  |  |  | ● |
| EDSS scoring | ● |  |  |  | ● |
| MSFC scoring | ● |  |  |  | ● |
| MRI | ● |  |  |  | ● |
| Drug accountability |  |  | ● |  | ● |
| Concomitant medication | ● | ● | ● | ● | ● |
| Adverse events |  | ● | ● | ● | ● |

Lab evaluation (s. 8.2.2):

1. Serum Chemistry, Serum lipids, Hematology, Creatine phosphokinase, Thyroid Profile
2. Immunology (2 serum-probes):

- Neutralizing Antibodies (Nab), Anti-MOG-, anti-MBP-Antibodies

# Activities

## Baseline visit (Day 1 visit)

- **The Screening visit is the final visit (month 15) of the SWABIMS-Study !!!**
- Before continuing the medication in the SWABIMS Follow up Study, the patient must give his/her written informed consent to participate.

### Procedures at baseline:

- Demographic data
- Inclusion and exclusion criteria
- Medical and surgical history
- MS history
- Medication history
- The contraceptive method used will be recorded, and if applicable, the age at menopause. Patients of childbearing potential will be instructed to discontinue study medication if they become pregnant and immediately notify the treating physician.
- Informed consent signature
- Vital signs
- Neurological examination by Evaluating Physician: EDSS
- MSFC scoring by Evaluating Physician or study nurse
- Physical examination
- Laboratory evaluations (Serum Chemistry, Serum lipids, Hematology, Urinalysis, Creatine phosphokinase, Thyroid Profile, Immunology (2 serum-probes stocks): Neutralizing Antibodies (Nab), Anti-MOG-, anti-MBP-Antibodies
- Pregnancy Test in women
- ECG
- MRI scan

## Treatment phase

The treatment phase begins at the Baseline visit (visit month 15 of the SWABIMS-Study) and terminates at months 12 visit.

### Month 6 visit (+/- 7 days)

- Physical examination
- Vital signs
- Drug accountability
- Concomitant medication
- Adverse events
- Laboratory testing:
  1. Serum Chemistry, Serum lipids, Hematology, Creatine phosphokinase, Thyroid Profile

### Months 12 Visit (+/- 7 days)

- Physical examination
- Vital signs
- Drug accountability (The unused study medication and used medication bottles will be collected).
- Concomitant medication use
- Adverse events: The patients will be queried orally regarding any AE they have experienced since the previous visit.
- Laboratory testing: Serum Chemistry, Serum lipids, Hematology, Urinalysis, Creatine phosphokinase, Immunology (2 serum-probes stocks): Neutralizing Antibodies (Nab), Anti-MOG-, anti-MBP-Antibodies
- MRI scan
- Neurological examination by Evaluating Physician (EDSS and MSFC)
- ECG

### Months 3 and 9 Visits (+/- 7 days)

- The patient will be called in order to collect information regarding concomitant medication an adverse events.

### Unscheduled Visits

There are 2 reasons for an unscheduled visit.

1. If during the center-initiated telephone call to the patient the patient believes that they are having a relapse or if a patient should call the study center with complaint of a relapse, then the patient will be instructed to come in for an unscheduled visit within 7 days.

- Physical examination with vital signs
- Relapse reporting
- EDSS
- Adverse Events

2. If during the treatment phase a patient complains of symptoms related to medication side effects that the Treating Physician thinks requires a visit, the patient will come for an unscheduled visit.

# STATISTICAL METHODS

## Statistical evaluation

### Methods of statistical analysis

The methodology described in the protocol represents the methods foreseen at the time of writing, but advances in medical or statistical understanding may necessitate amendments to the approach, which will be documented in the statistical analysis plan. Full details of the methodology, including handling of missing or spurious data, will be described in the statistical analysis plan. No person privy to unblinded data will participate in discussions concerning changes in the statistical analysis plan.

All statistical hypothesis tests will be two-sided tests performed at the 5% significance level. All confidence intervals will be two-sided 95% confidence intervals unless stated otherwise.

### Evaluable subjects and analysis population

All efficacy and safety analyses will be performed on the set of all randomized patients who took at least 1 dose of study medication, i.e., the Full Analysis Set (FAS).

### Study endpoints

**Primary Endpoint**

The primary efficacy endpoint is the proportion of patients in each treatment group with new T2 lesions after 12 months of treatment. The presence of new T2 lesions will be determined by comparing the results of the blind review of the MRI scans between study entry and Month 12.

### Statistical analyses

**Primary Endpoint**

The number and proportion of patients with new T2 lesions at month 12 will be summarised by treatment group. A Fisher’s Exact Test (or chi-squared test, if required) will be conducted to test for the unadjusted influence of treatment group.

The primary comparison between the groups will be performed using a logistic regression analysis. In addition to a term for treatment, the following terms will be included in the logistic regression model: number of T2 lesions at baseline, number of Gd-enhancing lesions at baseline, relapse rate at baseline, baseline EDSS score, time since diagnosis, sex. The results will be presented as odds ratios, and associated 95% confidence intervals and p-values.

The number of T2 lesions at Month 12 will also be analysed, using analysis of covariance (ANCOVA). In addition to a term for treatment the following covariates will be included in the model: number of T2 lesions at baseline, number of Gd-enhancing lesions at baseline, relapse rate at baseline, baseline EDSS score, time since diagnosis, sex.

**Secondary Endpoints**

The change in the number of Gd-enhancing lesions on T1-weighted images detected from baseline to Month 12, the change in total T2 hyperintense lesion volume from baseline to Month 12, the change in total EDSS and total MSFC from baseline to Month 12, the change in functional sub-scores of EDSS and MSFC from baseline to Month 12, cortical atrophy from baseline to Month 12 and the change in concentration of sieric levels of antibodies (anti-MOG and anti-MBP) will each be analysed using an ANCOVA model which will include terms for treatment group, baseline value (relevant to each endpoint), number of T2 lesions at baseline, number of Gd-enhancing lesions at baseline, relapse rate at baseline, baseline EDSS score, time since diagnosis, sex. If any of the normality assumptions underlying the ANCOVA model cannot be substantiated then a non-parametric test (Kruskal-Wallis) will be used.

The proportion of patients in each treatment group who remain relapse free after 12 months will be analysed using a Fisher’s Exact test. While the time to first relapse will be summarised using a Kaplan-Meier plot with patients who do not experience a relapse by the Month 12 being regarded as censored observations at month 12. Patients who drop-out of the study prior to month 12 will be regarded as censored at the time of withdrawal.

### Demographic and Baseline Characteristics

Demographic variables, such as age, race, and sex, and baseline characteristics, such as medical history, will be summarized by treatment group using descriptive statistics. For quantitative data, this will include the number of observations, the mean, standard deviation, median, maximum and minimum. Qualitative data will be summarised using frequency counts and percentages.

### Safety Analyses

A summary of the number of patients experiencing any adverse event and number of adverse events experienced by patients in each treatment group will be produced. This summary will also show the number of adverse events that were defined as serious and number of patients experiencing SAEs.

The number of patients experiencing each adverse event will be shown using the cross-classification of preferred term (within body system class) by most severe intensity. All adverse events occurring in more than 5% of either treatment group will be summarised, giving the incidence of each event by preferred term.

Serum chemistry and hematology measurements will be listed, with any out-of-range values being flagged as high or low according to their age specific laboratory normal ranges. Values will be summarised by visit using descriptive statistics and shift tables will be produced for each parameter showing the changes with respect to normal laboratory ranges from the baseline visit to Month 12 visit.

# ETHICS

## Ethical conduct of the study

The planning and conduct of this clinical study are subject to national laws. Only when all of the requirements of the appropriate regulatory authority have been fulfilled will the study begin. The study will be conducted in accordance with the ethical principles of the Declaration of Helsinki and the ICH-GCP Guidelines of 17 Jan. 1997(G.U. n 191, 18 Ago 1997) and with the appropriate national regulations.

## Written Patient information and informed consent form

The investigator will explain the nature of the study, its purpose and associated procedures, the expected duration and the potential benefits and risks of participation to each patient prior to his/her entry into the study (i.e. before examinations and procedures associated with selection for the study are performed). The investigator will provide the patient with an IRB/IEC-approved patient information and informed consent form. Each patient will have ample opportunity to ask questions and will be informed about the right to withdraw from the study at any time without any disadvantage and without having to provide reasons for this decision.

Following this informative discussion a patient will be asked if he/she is willing to personally sign and date a statement of informed consent. Only if the patient voluntarily agrees to sign the informed consent statement, and has done so, may he/she enter the study. The patient will receive a copy of his/her signed and dated form.

## Confidentiality

The protocol data provided for this study to Ethics committee and investigators staff are confidential. Investigators must assure anonymity to the patients. For this reason the documents sent to the sponsor will be identify only with initial letters of patient. However the investigator must archive the listing of the identities of patients involved in the study.

# Reference list

1. Sadovnick AD, Ebers GC.

Epidemiology of multiple sclerosis: a critical overview.
Can J Neurol Sci. 1993 Feb;20(1):17-29

1. Weinshenker B, Bass B, Rice G, Noseworthy J, Carriere W, Baskerville J, et al. The natural history of multiple sclerosis: a geographically based study. I. Clinical course and disability. Brain 1989;112:133-146
2. Confavreux C, Aimard G, Devic M

Course and prognosis of multiple sclerosis assessed by the computerized data processing of 349 patients.
Brain. 1980 Jun;103(2):281-300.

1. Lublin FD, Reingold SC.

Defining the clinical course of multiple sclerosis: results of an international survey. National Multiple Sclerosis Society (USA) Advisory Committee on Clinical Trials of New Agents in Multiple Sclerosis.
Neurology. 1996 Apr;46(4):907-11.

1. IFNB Multiple Sclerosis Study Group 1993

Interferon beta-lb is effective in relapsing-remitting multiple sclerosis. I. Clinical results of a multicenter, randomized, double-blind, placebo-controlled trial. Neurology; 1993 43; 655-661.

1. Paty DW, Li DK. 1993

Interferon beta-lb is effective in relapsing-remitting multiple sclerosis. I. Clinical results of a multicenter, randomized, double-blind, placebo-controlled trial.

Neurology. Apr;43(4):662-7

1. LaRosa JC et al. 1999

Effect of statins on risk of coronary disease. A meta-analysis of randomized controlled trials.
JAMA 282:2340-2346

1. Black DM et al. 1998

An overview of the clinical safty profile of atorvastatin, a new HMG-CoA reductase inhibitor.

Arch Intern Med 158: 577-584

1. Gotto et al 2003

Safety and statin therapy: reconsidering the risks and benefits.
Arch Intern Med. Mar 24;163(6):657-9.

1. Takemoto M, Liao JK. 2001

Pleiotropic effects of 3-hydroxy-3-methylglutaryl coenzyme a reductase inhibitors.
Arterioscler Thromb Vasc Biol. Nov;21(11):1712-9

1. Youssef S, Stuve O, Patarroyo JC, Ruiz PJ, Radosevich JL, Hur EM, Bravo M, Mitchell DJ, Sobel RA, Steinman L, Zamvil SS 2002

The HMG-CoA reductase inhibitor, atorvastatin, promotes a Th2 bias and reverses paralysis in central nervous system autoimmune disease.

Nature. Nov 7;420(6911):78-84.

1. Stanislaus R, Singh AK, Singh I. 2001

Lovastatin treatment decreases mononuclear cell infiltration into the CNS of Lewis rats with experimental allergic encephalomyelitis.
J Neurosci Res. Oct 15;66(2):155-62

1. Aktas O, Waiczies S, Smorodchenko A, Dorr J, Seeger B, Prozorovski T, Sallach S, Endres M, Brocke S, Nitsch R, Zipp F 2003

Treatment of relapsing paralysis in experimental encephalomyelitis by targeting Th1 cells through atorvastatin.

J Exp Med. Mar 17;197(6):725-33.

1. Kwak B, Mulhaupt F, Myit S, Mach F 2000

Statins as a newly recognized type of immunomodulator.

Nat Med. Dec;6(12):1399-402

1. Greenwood J, Walters CE, Pryce G, Kanuga N, Beraud E, Baker D, Adamson P. 2003

Lovastatin inhibits brain endothelial cell Rho-mediated lymphocyte migration and attenuates experimental autoimmune encephalomyelitis

FASEB J. May;17(8):905-7

1. Neuhaus O, Strasser-Fuchs S, Fazekas F, et al. 2002

Statins as immunomodulators.

Neurology;59:990-997

1. Vollmer T, Key L, Durkalski V, Tyor W, Corboy J, Markovic-Plese S, Preiningerova J, Rizzo M, Singh I. 2004
   Oral simvastatin treatment in relapsing-remitting multiple sclerosis.
   Lancet. May 15;363(9421):1607-8
2. Vaughan CJ, Delanty N. Neuroprotective properties of statins in cerebral ischemia and stroke. Stroke. 1999 Sep;30(9):1969-73.
3. Vaughan CJ, Delanty N, Basson CT. Do statins afford neuroprotection in patients with cerebral ischaemia and stroke? CNS Drugs. 2001;15(8):589-96
4. Blume C, Sabuda-Widemann D, Pfeilschifter J, Plum J, Schror K, Grabensee B, Beck KF. Cerivastatin inhibits proliferation of interleukin-1beta-induced rat mesangial cells by enhanced formation of nitric oxide.Eur J Pharmacol. 2004 Feb 6;485(1-3):1-10.
5. Guzik TJ, Korbut R, Adamek-Guzik T. Nitric oxide and superoxide in inflammation and immune regulation. J Physiol Pharmacol. 2003 Dec;54(4):469-87
6. Cannon CP, Braunwald E, McCabe CH, Rader DJ, Rouleau JL, Belder R, Joyal SV, Hill KA, Pfeffer MA, and Skene AM for the Pravastatin or Atorvastatin Evaluation and Infection Therapy – Thrombolysis in Myocardial Infarction 22 Investigators. Comparasion of intensive and moderate lipid lowering with statins after acute coronary syndrome. N Engl J Med. 2004; 350 (15).
7. Newman CB, Palmer G, Silbershatz H, Szarek M. Safety of atorvastatin derived from analysis of 44 completed trials in 9,416 patients. Am J Cardiol. 2003 ;92(6):670-6
8. Stanislaus et al. 1999

Amelioration of experimental allergic encephalomyelitis in Lewis rats by lovastatin

Neuroscience letters 269: 71-

1. Sena A et al. 2003

Therpeutic potential of lovastatin in multiple sclerosis

J Neurol 250: 754-755

1. Hafler DA. Multiple sclerosis. J Clin Invest 2004; 113(6):788-794.
2. Sakaguchi S, Sakaguchi N, Asano M, Itoh M, Toda M. Immunologic self-tolerance maintained by activated T cells expressing IL-2 receptor alpha-chains (CD25). Breakdown of a single mechanism of self-tolerance causes various autoimmune diseases. J Immunol 1995; 155(3):1151-1164.
3. Yamazaki S, Iyoda T, Tarbell K, Olson K, Velinzon K, Inaba K et al. Direct expansion of functional CD25+ CD4+ regulatory T cells by antigen-processing dendritic cells. J Exp Med 2003; 198(2):235-247.
4. Yilmaz A, Reiss C, Tantawi O, Weng A, Stumpf C, Raaz D et al. HMG-CoA reductase inhibitors suppress maturation of human dendritic cells: new implications for atherosclerosis. Atherosclerosis 2004; 172(1):85-93.
5. Wandinger KP, Sturzebecher CS, Bielekova B, Detore G, Rosenwald A, Staudt LM, McFarland HF, Martin R., Complex immunomodulatory effects of interferon-beta, in multiple sclerosis include the upregulation of T helper 1-associated marker genes, Ann Neurol. 2001 Sep;50(3):349-57.

# List of Abbreviations

| ACTH | Adrenocorticotropic hormone |
| --- | --- |
| AE | adverse event |
| ALAT | alanine aminotransferase |
| ANC  Anti-MBP  anti-MOG  ASAT  BOD | absolute neutrophil count  antibodies against myelin basic protein  antibodies against myelin oligodendrocyte glycoprotein  aspartate aminotransferase  burden of disease |
| BRM | biologic response markers |
| CHO | Chinese hamster ovary |
| DC  CIITA  CNS  CPK | dendritic cells  class II transactivator  central nervous system  creatine phosphokinase |
| CRA  CRF  CRO | Clinical Research Associate  Case Report Form  Contract Research Oranization |
| EAE | Experimental Allergic Encephalomyelitis |
| EDSS | Expanded Disability Status Scale |
| ECG  eNOS  iNOS  eod | Electrocardiogram  Endothelial nitric oxide synthase  Inducible nitric oxide synthase  every other day |
| EOS  EP  FAS | end of study  Examining physician  Full Analysis Set |
| FDA  FOV | Food and Drug Administration  Field of view |
| GCP  Gd  GGT | Good Clinical Practice  Gadolinium  gamma-glutamyltranspeptidase |
| Hb  HCG  HDL  HMG-CoA | Hemoglobin  human chorionic gonadotropin  High-density lipoprotein  3-hydroxy-3-methylglutaryl-coenzyme A |
| ICH  IDL | International Committee on Harmonization  Intermediate-density lipoprotein |
| IEC  IFN  IL | Independent Ethics Committee  Interferon  Interleukin |
| IM | Intramuscular |
| IMB | Independent Monitoring Board |
| IRB  ITT | Institutional Review Board  Intent to treat |
| IU  iv | International units  intravenous |
| LDL  LFA-1 | Low- density lipoprotein  Leukocyte Function Antigen-1 |
| MHC | major histocompatibility complex |
| MIU  MMP-9 | million international units  matrix metalloproteinase 9 |
| MRI  MRI-AC | magnetic resonance imaging  magnetic resonance imaging – Analysis Center |
| MS | multiple sclerosis |
| MSFC | multiple sclerosis functional composite score |
| Nabs | neutralizing antibodies |
| NSAID  PBMC  PP-MS | Nonsteroidal anti-inflammatory drug  Peripheral Blood Mononuclear Cells  Primary progressive multiple sclerosis |
| QOL  RBC  RR-MS | Quality of Life  Red blood cells  Relapsing-remitting multiple sclerosis |
| SAE | serious adverse event |
| sc  SP-MS  STAT  TG  TGF | subcutaneous  Secondary progressive multiple sclerosis  Signal Transducer and Activator of Transcription  Triglycerides  Transforms growth factor |
| TNF  Th1  Th2  TP  TSH  VLDL | tumor necrosis factor  CD4+ helper T cells type 1  CD4+ helper T cells type 2  Treating physician  Thyroid-stimulating hormone  Very-low-density lipoprotein |
| WBC | white blood cells |

# Appendix

## Magnetic Resonance Imaging

### Overview

In this multicenter trial, scans will be assessed centrally for number and volume of lesions and for normalized brain volume. For central assessment it is of vital importance that all scans adhere to the standardized MRI protocol, which might be different from the standards otherwise used.

T1-weighted (after Gadolinium injection) and dual-echo MRI scans of the brain will be

performed at screening in order to check MRI inclusion criteria (the inclusion criteria will be assessed locally at the MRI site); this scan will also serve as the baseline scan.

Each center should use the same scanner for each patient for the entire duration of the study. All scanners should have at least 1.0 Tesla magnetic field strength. The following institutions will be involved in the central assessment of the MRI images:

The MRI Analysis Center (MRI- AC) at Bern will perform the entire MR image analysis.

Quality control of the images and data conversion if necessary will be performed by

the sponsor, or by the MRI Core Lab.

### Scanning requirements

###### Procedure

Given the importance of careful repositioning for serial MRI lesion counting and load assessments, great care should be taken when positioning the patients in the scanner and head holder. The following rules apply to all centers:

- Check that the patient can successfully undergo MRI examination
- Enter the patient data into the scanner console, using:
- Patient initials (first letter of given name, first letter of surname)
- Study number, center number, patient number
- Date of birth (dd / mm / yyyy)
- Sex (m/f)
- Scan number (use progressive numbers starting from –1 for the screening scan. If a scan is rejected and has to be repeated the letter R will be added to the scan number, *e.g*.
- 1R1 for the first repetition of the screening scan, -1R2 for the second repetition of the screening scan)
- Explain the scanning procedure to the patient and position her/him in the scanner in the most comfortable position.
- Insert an intravenous needle in the patient’s arm and connect it with a long-line catheter to a drip-infusion of saline; for contrast injection the operator will be able to use the long-line without moving the patient table from the scanner.
- Position the patient’s head and align it in the machine using land-marking devices provided with most of the equipment.
- Position the patient’s eyebrows at the center of the coil and make sure that the horizontal light beam runs over the eyebrows and as close as possible to the line marking the coil center.
- Position the nose along the Z axis of the scanner, *i.e*. make sure that the vertical light beam runs over the nose.
- Use foam cushions and strips to hold the patient’s head in the set position.
- Move the patient into the scanner.

### Imaging

The following order for sequences has to be kept:

1. T1-weighted axial scout

2. T1-weighted coronal scout

3. T1-weighted sagittal scout

4. Rapid T1-weighted SE or T2-weighted FSE/TSE axial (for repositioning check)

5. T2-weighted FSE/TSE

6. T1-weighted SE post gadolinium

Sequences 1 to 4 are performed to obtain reference images for patient repositioning.

### Sequence parameters, T1 axial scout:

- – TR: 100
- – TE: 10-20
- – Slice number: 3
- – Slice thickness: 5mm
- – Orientation: axial
- – Field of view (FOV): 210-230 mm
- – Matrix: 128 x 256
- – Inter-slice gap: 5 mm
- – Number of acquisitions: 1
- – Phase encoding: L > R

### Sequence parameters, T1 coronal scout:

Using sequence 1 as a reference, make a coronal scout, parallel to the brain transverse diameter and depicting the midline of the brain best (*i.e*. at the level of the brainstem); the acquisition parameters will be the following:

- – TR: 100
- – TE: 10-20
- – Slice number: 1
- – Slice thickness: 5 mm
- – Orientation: coronal
- – Field of view (FOV): 210-230 mm
- – Matrix: 128 x 256
- – Number of acquisitions: 1
- – Phase encoding: L > R

### Sequence parameters, T1 sagittal scout:

From the coronal scout make a sagittal scout image, aligned with the falx cerebri and other midline structures, following these parameters:

- – TR: 500-650
- – TE: 10-20
- – Slice number: 1
- – Slice thickness: 5 mm
- – Orientation: sagittal
- – Field of view (FOV): 210-230 mm
- – Matrix: 128 x 256
- – Number of acquisitions: 1-3
- – Phase encoding: A > P

### T1-weighted images to check correct positioning:

On the sagittal scout image previously obtained, position the axial image group, placing the center of the slice group at the inferior borders of the corpus callosum genu and splenium. The slice group will be positioned to include the whole brain from the vertex to the level of the foramen magnum. Save the localizer image with the slices shown on it, to achieve the same slice positioning on subsequent scans; then make a rapid series of either T1-weighted or T2-weighted images (according to site preference) in order to check the correct positioning of the slices. The following parameters should be used:

- – TR: 500-600
- – TE: 10-20
- – Slice number: 22
- – Slice thickness: 3 mm
- – Orientation: axial
- – Field of view (FOV): 250 mm
- – Matrix: 140 x 256
- – Inter-slice gap: 3 mm
- – Series: interleaved
- – Number of acquisitions: 1
- – Phase encoding: L > R

### T2-weighted images to check the correct positioning:

For T2-weighted images, a fast spin echo (or turbo spin echo) sequence should be used with the following parameters:

- TR: 1800-2800
- TE: either single-echo or dual-echo (according to site preference):TE first echo: 30-50; TE second echo: 60-100
- ETL: according to site preference
- Slice number: 22
- Slice thickness: 3 mm
- Orientation: axial
- FOV: 250 mm
- Matrix: approximately 140 x 256.The number of phase-encode lines will depend on the exact sequence used
- Inter-slice gap: 3 mm
- Series: interleaved
- Number of acquisitions: 1
- Phase-encoding: L > R

**Note:** This first series of T1- or T2 axial images is only performed at the baseline scan and then used as a positioning reference for subsequent serial scans.

### Dual-echo images:

Record the slice positioning parameters (shift and rotation angle) of this series and use them for the subsequent series of the same scan (not necessary if the scanner has a repeat or history function). If possible, to speed up the examination, use a rectangular 3/4 FOV (*i.e*., reduce the phaseencoding matrix by 25%) for all subsequent series of axial images. With 3/4 FOV, the FOV is 187.5 in L > R direction and 250 mm in A > P direction; matrix is 256 (read) x 192 (phase). The scanning procedure is resumed with two interleaved series of fast or turbo spin-echo dual echo sequences. Conventional spin-echo sequences are acceptable if fast imaging is not available. The image parameters are as follows:

- TR: 2200-3000
- TE: 15-50/80-120
- ETL: 4-6
- Slice number: 22
- Slice thickness: 3 mm
- Orientation: axial
- FOV: 250 mm
- Matrix: 256 x 256 (192 x 256 if RECFOV)
- Inter-slice gap: 3 mm
- Series: interleaved
- Number of acquisitions: 1
- Phase encoding: L > R
- Presaturation slab/flow compensation: Yes

The axial presaturation slab (50 to 80 mm) must be positioned inferior to the slice group to suppress flow-related artifacts. The second series is performed with the slice position shifted 3 mm caudally compared with the first one, so that when both series are combined, the whole brain is covered. Some General Electric and Philips scanners allow the two separate acquisitions (each of 22 slices) to be performed sequentially and automatically from a single prescription. If the scanner used allows the acquisitions to be split automatically in this way, then this should be done.

Perform a bolus injection of high concentrated gadolinium (i.e. 1.0 mol/L, gadobutrol (gadolinium-DO3A-butriol) using the intravenous long-line and without moving the patient from the scanner. Use contrast at a dose of 0.2 mmol of gadolinium per kilogram of body weight (*i.e*. 0.2 ml/kg). After a post-injection delay of 5 minutes, complete the scanning with postgadolinium T1-weighted SE images. The following parameters should be used.

### T1-weighted post-gadolinium images:

T1-weighted sequences of MRI scans will be performed with concentrated (1 mol/L) gadobutrol at a dose of 0.2 mmol of gadolinium per kilogramm of body weight.

- TR: 550-700 (Keep the TR within the range and optimize the signal to noise ratio; *i.e*. the maximum echo time and the lowest bandwidth, consistent with the TR range)
- TE: 10-20
- Slice number: 22
- Slice thickness: 3 mm
- Orientation: axial
- Field of view (FOV): 250 mm
- Matrix: 256 x 256 (192 x 256 if RECFOV)
- Inter-slice gap: 3 mm
- Series: interleaved
- Number of acquisitions: 2
- Phase encoding: L > R
- Presaturation slab/flow compensation: yes

The second series of images will be positioned to fill the gaps of the first one. Once the two acquisitions are completed, check the image consistency in terms of both correct repositioning (when compared with “fast” T1) and covering the whole brain as indicated above. In case of spoiled post-contrast T1-weighted images due either to patient movement artifacts, or to a change in slice position of six mm or more (*i.e*. > 2 slice thickness compared to baseline scan), post-contrast T1-weighted sequence should be repeated no later than 20 minutes after gadolinium-DTPA injection. When the MRI screening scan is complete, it is the responsibility of the MRI investigator to assess whether the abnormalities present on the MRI scans are compatible with MS. If any other neurological condition is suspected on the basis of the MRI data, the MRI investigator must inform the clinical investigator, who will decide whether the screening process should be stopped. The MRI investigator will also inform the clinical investigator if pathological changes other than those associated with MS are noticed in follow-up MRI scans. Any further discussion of MRI follow-up scans between the MRI investigator and the clinical investigator is strictly prohibited.

### Electronic data

Each participating center is to send a copy of the MRI digital images immediately after image acquisition to department of neuroradiology Bern. As a general guideline the following is a list, ordered by preference, of the acceptable media:

1. CD-ROM

2. Electronic tapes: 4 mm DAT or 8 mm video 1/4” tape DLT

3. Network transfer ftp

4. Optical disk

Tapes, optical disks and CD-ROMs are to be sent via courier to the MRI Core Lab. Data sent by network transfer should also be sent immediately and the MRI Core Lab notified of the transfer, with a detailed list of the data sent. Whatever the means of transfer chosen, all electronic data of the scan, including scout images must be sent. Electronic data should be sent in DICOM format.

### Archiving

Each center has to keep electronic backup copies of all MRI data at the local site (*i.e*. two

electronic backups are needed, one for local storage and one to be sent to the MRI-AC).

### MRI scan analysis

Two experienced observers will be in charge of lesion identification and counting. All scans from a given patient will be evaluated by the same observer. Doubtful cases will be assessed by the observers by consensual agreement. The readers were masked to clinical events or treatment.

A single enhancing lesion is defined as an area of enhancement seen on a given 3 mm axial image, which is referable neither to normally enhanced structures, nor to contrast migration within vessels. T2-weighted FSE/TSE images will be used as reference for analysis. A single T2 lesion is defined as an area of increased signal on a given 3 mm axial image, which should be seen on both T2- and proton density-weighted images and which is not referable to normally hyperintense structures. Lesions that changed during the study were categorized as new, enlarging, or recurrent. New T2 lesions are those which appear in areas where on the previous scan no abnormality was detected.

Lesion load will be assessed by trained technicians. The same technician will measure abnormalities on the scans from the same patients throughout the entire study. The image analysis will be performed using a semi-automated segmentation technique based on local thresholding.

### MRI scan quality control

The quality of scans will be reviewed by the MRI Core Lab. Within two working days of receiving the scan, the MRI Core Lab will fax the results of scan consistency to the participating centers.

If the quality of any image is unsatisfactory, the scans will be rejected. Rescanning will be

allowed and should be performed not later than 14 days after the original scans have been

obtained. Repositioning will be considered acceptable when less than a 2-slice difference

between MRI sessions exists for recognizable landmarks on the axial slices.

## Acceptable ranges for laboratory findings

- Hemoglobin (Hb) ≥9.0 g/dL
- White blood cell count (WBC) ≥2,500 and ≤15,000/mm^3^
- Absolute neutrophil count (ANC) ≥750/mm^3^
- Platelet count ≥100,000/mm^3^
- Absolute lymphocyte count ≥750/mm^3^
- Alanine aminotransferase (ALAT) ≤3 times the upper limit of normal
- Total bilirubin < 3.0 mg/dL
- Creatinine < 2.0 mg/dL at baseline
- CPK < 3 times upper limit of the normal range at baseline
- TSH < 6.75 mU/l

# Patient information and consent

## Patient Information

**PatientInneninformation**

**Schweizer randomisierte, multizentrische, Auswerter-verblindete, parallelarm Studie, die bei Patienten mit schubförmig-remittierender Multipler Sklerose die Wirksamkeit, Sicherheit und Verträglichkeit der Kombinationstherapie von Betaferon 250 μg (8 MIU) subkutan gegeben jeden zweiten Tag mit Atorvastatin 40 mg/Tag mit der Wirksamkeit, Sicherheit und Verträglichkeit einer Betaferon-Monotherapie 250 μg (8 MIU), subkutan gegeben jeden zweiten Tag, vergleicht – SWABIMS Follow up Studie**

Efficacy, safety and tolerability of Atorvastatin 40 mg in patients with relapsing-remitting multiple sclerosis treated with Interferon-beta-1b - SWABIMS Follow up Study

**Multi-center, randomized, rater-blinded, parallel-group-study in Switzerland**

**SWiss Atorvastatin and Interferon-Beta 1b Trial In Multiple Sclerosis**

**“SWABIMS-Follow up-Study”**

##### Sehr geehrte Patientin,

##### Sehr geehrter Patient,

Sie haben bereits an der SWABIMS-Studie teilgenommen. Im Rahmen dieser Studie haben Sie 3 Monate jeden 2. Tag Interferon-beta-1b subkutan injiziert. Anschliessend wurden Sie zufällig einer der folgenden Behandlungsgruppen zugeordnet (= randomisiert).

1. Sie haben weiterhin jeden 2. Tag Interferon-beta-1b subkutan für 12 Monate injiziert oder
2. Sie haben weiterhin jeden 2. Tag Interferon-beta-1b subkutan injiziert und zusätzlich Atorvastatin 40 mg/Tag p.o. (als Tablette) für 12 Monate eingenommen.

Nach insgesamt 15 Monaten Behandlungsdauer werden die SWABIMS Studie beenden.

**1 Informationen zur SWABIMS-Studie**

In die SWABIMS-Studie konnten bisher mehr als 70 der geplanten 80 Patienten eingeschlossen werden.

Die Studie konnte bisher ohne Komplikationen durchgeführt werden, zu schwerwiegenden Nebenwirkungen ist es nicht gekommen. Die Studienmedikamente sowie die verschiedenen Untersuchungen wurden somit insgesamt gut vertragen.

Ergebnisse der Studie liegen aktuell nicht vor, da die Studie noch nicht beendet ist. Somit können noch keine Aussagen über den Einfluss der Behandlungen auf Ihre Erkrankung (Multiple Sklerose) gemacht werden.

Aufgrund des komplikationslosen Verlaufes wird die SWABIMS-Studie zur weiteren Informationsgewinnung über die Therapien in der **„SWABIMS-Follow up Studie“** verlängert.

**2 Informationen zur „SWABIMS-Follow up-Study“**

Mit der „SWABIMS-Follow up-Studie“ wird die SWABIMS-Studie um weitere 12 Monate fortgeführt.

Dabei werden die Behandlungsgruppen nicht verändert. Das bedeutet, dass jeder Teilnehmer unverändert die Medikamente einnimmt, die er während der SWABIMS-Studie eingenommen hat.

Lediglich die Anzahl der Visiten mit den jeweiligen Untersuchungen wird angepasst.

So findet alle 6 Monate eine Visite statt. Dabei werden jeweils Befragungen, klinische Untersuchungen, Untersuchungen im Blut sowie nach 12 Monaten Aufnahmen vom Kopf im Magnet-Resonanz-Tomographen durchgeführt. Die Visiten unterscheiden sich somit nicht von den Visiten während der SWABIMS-Studie. Die genauen Aktivitäten während der „SWABIMS-Follow up-Studie“ entnehmen Sie bitte der Tabelle 1.

Diese Visiten werden zur Überprüfung der Studienmedikamente und ihre Wirkung/Nebenwirkung, also zur Sicherheit des Teilnehmers durchgeführt. Des Weiteren werden für die Studie wichtige Daten erhoben.

Ausser in Notfällen dürfen Sie sich während der klinischen Prüfung einer anderen medizinischen Behandlung nur im Einvernehmen mit dem Prüfarzt unterziehen.

Jede Verschlechterung des Gesundheitszustandes während der Teilnahme an der Studie müssen Sie dem behandelnden Prüfarzt unverzüglich mitteilen.

**Tabelle 1: Aktivitäten während der „SWABIMS-Follow up-Studie“:**

| **Aktivitäten:** | **Tag 1** | **Monat 3**  **Telefon** | **Monat 6** (+/- 7 Tage) | **Monat 9**  **Telefon** | **Monat 12** (+/- 7 Tage) |
| --- | --- | --- | --- | --- | --- |
| Schriftliche Einverständniserklärung | ● |  |  |  |  |
| Prüfung der Ein- und Auschlusskriterien | ● |  |  |  |  |
| Persönliche Krankheitsgeschichte | ● |  |  |  |  |
| Demographische Daten | ● |  |  |  |  |
| Körperliche Untersuchung | ● |  | ● |  | ● |
| Vitalparameter | ● |  | ● |  | ● |
| EKG (Elektrokardiogramm) | ● |  | ● |  | ● |
| Medikamentenanamnese | ● |  |  |  |  |
| Laboruntersuchung | ● |  | ● |  | ● |
| Schwangerschaftstest | ● |  |  |  |  |
| EDSS-Test | ● |  |  |  | ● |
| MSFC-Test | ● |  |  |  | ● |
| MRI (Magnetresonanztomographie des Gehirns) | ● |  |  |  | ● |
| Medikamentenüberprüfung |  | ● | ● | ● | ● |
| Unerwünschte medizinische Ereignisse |  | ● | ● | ● | ● |

Die Baseline Visite der „SWABIMS-Follow up-Studie“ entspricht der letzten Visite (Monat 15) der SWABIMS-Studie. Im Rahmen der SWABIMS-Follow up-Studie sind somit nur 2 weitere Visiten geplant. In Monat 3 und 9 werden Sie telefonisch über Medikamente und unerwünschte Nebenwirkungen befragt.

**Ansonsten entsprechen die allgemeinen Regeln der „SWABIMS-Follow up-Studie“ exakt denen der ursprünglichen SWABIMS-Studie.**

**3 Ziele der „SWABIMS-Follow up-Studie“**

Das Ziel der „SWABIMS Follow up-Studie“ ist es, die Wirksamkeit, Sicherheit und Verträglichkeit der Kombinationstherapie von Betaferon 250 μg (8 MIU, jeden zweiten Tag subkutan gegeben) und Atorvastatin 40 mg/Tag bei Patienten mit schubförmiger Multipler Sklerose im Vergleich zur Betaferon-Monotherapie 250μg (8 MIU), (subkutan gegeben jeden zweiten Tag) **über einen verlängerten Zeitraum von 12 Monaten zu untersuchen**.

Ein möglichst langer Beobachtungszeitraum ist bei Langzeittherapien wie bei der Multiplen Sklerose sehr wichtig, da nur so die Wirkungen und Nebenwirkungen nach mehrjähriger Behandlungsdauer erforscht werden können.

**4 Freiwilligkeit der Teilnahme**

Auch die Teilnahme an der „SWABIMS Follow up-Study“ ist freiwillig. Wenn Sie auf die Teilnahme an dieser Studie verzichten, haben Sie keine Nachteile für Ihre weitere medizinische Betreuung zu erwarten. Das gleich gilt, wenn Sie Ihre dazugegebene Einwilligung zu einem späteren Zeitpunkt widerrufen. Diese Möglichkeit haben Sie jederzeit. Einen allfälligen Widerruf Ihrer Einwilligung bzw. den Rücktritt von der Studie müssen Sie nicht begründen.

**5 Pflichten des Studienteilnehmers**

Als Studienteilnehmer sind Sie verpflichtet, den medizinischen Anweisungen Ihres Prüfarztes zu folgen und sich an den Studienplan zu halten, Ihren Prüfarzt genau über den Verlauf der Erkrankung und festgestellte unerwünschte Wirkungen zu informieren, Ihren Prüfarzt über die gleichzeitige Behandlung bei einem anderen Arzt und über die Einnahme von Arzneimitteln (von einem Arzt verordnete und selbstständig und ohne ärztliches Rezept gekaufte) aufzuklären. Während der Studie gibt es bestimmte Medikamente, die Sie weiterhin einnehmen können, und andere die nicht erlaubt sind. Über die Behandlung der Schübe entscheidet Ihr Neurologe. **Es ist sehr wichtig**, dass Sie sofort Ihren behandelnden Studienarzt benachrichtigen, falls Sie der Meinung sind, dass neue Symptome Ihrer Erkrankung aufgetreten sind. Ihr Studienarzt wird Sie dann zu einem ausserplanmässigen Besuch aufbieten und eine komplette neurologische Untersuchung und Beurteilung möglicherweise mit Zusatzuntersuchungen und Behandlungen durchführen.

Im Rahmen der Studie muss eine Schwangerschaft verhindert werden. Weibliche Studienteilnehmer müssen während der Studie eine zuverlässige Methode der Verhütung anwenden (Pille, mechanische Verhütungsmethode, wie z.B. Diaphragma, Spirale etc.). Patientinnen, die während der Studie schwanger werden, müssen Ihren Arzt umgehend informieren (und dürfen nicht weiter an der Studie teilnehmen). Frauen in der Stillzeit sind von einer Studienteilnahme ausgeschlossen.

**6 Alternative Behandlungsmethoden**

Wenn Sie nicht an dieser Studie teilnehmen wollen, bestehen möglicherweise alternative Behandlungsmöglichkeiten in Form einer immunmodulatorischen Therapie mit Interferon-beta-1b (Betaferon®) jeden zweiten Tag subkutan (der Kontrollgruppe der Studie entsprechend), Interferon-beta-1a 3x/Woche (Rebif®) unter die Haut, Interferon-beta-1a 1x/Woche (Avonex) intramuskulär oder mit Glatiramer-acetat (Copaxone®) jeden Tag unter die Haut zur Verfügung. Bei rasch progredienter Verschlechterung stünde alternativ auch eine Behandlung mit Mitoxantron, einem Chemotherapeutikum zur Verfügung. Ihr Arzt wird Sie über diese Möglichkeiten und Unterschiede zur Studienmedikation aufklären. Auch während der Studie werden Sie bei neuen Erkenntnissen bezüglich Therapien umgehen informiert.

**7 Nutzen für die Teilnehmer**

In dieser Studie erhalten die Teilnehmer auf jeden Fall ein aktives Medikament, das in dieser Form auch als Standardtherapie zu erhalten ist. Niemanden wird ein Scheinmedikament (Placebo) verabreicht. Zusätzlich profitieren die Studienteilnehmer von einer intensiveren klinischen-, laborchemischen- und radiologischen Kontrolle im Vergleich zu ausserhalb der Studie behandelten Personen.

Es könnte jedoch sein, dass Sie keinen direkten Nutzen haben, und es gibt keine Garantie, dass sich Ihre Erkrankung durch eine Studienteilnahme verbessert. Dank Ihrer Studienteilnahme können die Ergebnisse auch anderen Personen zugute kommen.

8 Risiken und Unannehmlichkeiten

Die Studie wird unter sorgfältig kontrollierten Bedingungen durchgeführt. Jedoch kann jegliche medizinische Behandlung zu unerwünschten Nebenwirkungen führen. Deshalb ist es sehr wichtig, dass Sie alle Änderungen Ihres gesundheitlichen Zustands schnellstmöglich Ihrem behandelnden Neurologen berichten.

Interferon-beta-1b (Betaferon®) wurde seit der Erstzulassung 1993 bei über 115.000 MS-Patienten eingesetzt. Atorvastatin (Sortis) wird ebenfalls seit vielen Jahren in Studien bzw. in routinemässiger Behandlung der Hypercholesterinämie eingesetzt. Die Nebenwirkungsprofile dieser Medikamente sind somit bestens bekannt. Falls indiziert werden auch heute schon beide Medikamente zusammen verordnet. Kontraindikationen für die gleichzeitige Anwendung beider Präparate bestehen nicht. Auch in kleineren, die Verträglichkeit der Kombinationstherapie prüfenden Studien zeigte sich eine gute Verträglichkeit.

Durch die Injektionslösung, die Ihnen vor der MRT-Aufnahme gespritzt wird, könnte es zu einem Unwohlsein kommen. Es ist bekannt, dass diese Lösung in seltenen Fällen allergische Reaktionen auslösen kann. Bei neuen Erkenntnissen werden Sie umgehend informiert.

**9 Vertraulichkeit der Daten**

In dieser Studie werden persönliche Daten von Ihnen erfasst. Diese Daten werden anonymisiert. Sie sind nur Fachleuten zur wissenschaftlichen Auswertung zugänglich. Die Kantonale Ethikkommission und die Mitglieder der zuständigen Behörden können Einsicht in die Originaldaten verlangen. Während der ganzen Studie und bei den erwähnten Kontrollen wird die Vertraulichkeit strikt gewahrt. Ihr Name wird in keiner Weise in Rapporten oder Publikationen, die aus der Studie hervorgehen, veröffentlicht.

**10 Versicherungsschutz**

Der Prüfer ersetzt Ihnen Schäden, die Sie gegebenenfalls im Rahmen des klinischen Versuchs erleiden. Zu diesem Zweck hat das Spital eine Versicherung abgeschlossen. Stellen Sie während oder nach dem klinischen Versuch gesundheitliche Probleme oder andere Schäden fest, so wenden Sie sich bitte an den verantwortlichen Arzt. Er wird für Sie die notwendigen Schritte einleiten. Sie dürfen die Versicherungsbedingungen jederzeit beim Prüfarzt einsehen.

**11 Vergütung von Auslagen des Studienteilnehmers**

Bis auf die Interferon-beta-1b (Betaferon®)Therapie sind die in dieser Information erwähnten Untersuchungen kostenlos. Weder Ihnen noch Ihrer Krankenkasse entstehen im Zusammenhang mit Ihrer Teilnahme zusätzliche Kosten.

**12 Kontaktpersonen**

Bei Unklarheiten, Notfällen, unerwarteten oder unerwünschten Ereignissen, die während der Studie oder nach deren Abschluss auftreten, können Sie sich an die untenstehenden Kontaktpersonen wenden:

Prof. Dr. med. Heinrich Mattle

Dr. med. Christian Phillip Kamm

Multiple Sklerose Sprechstunde

Neurologische Poliklinik des Inselspitals Bern

3010 Bern
Tel: 031-6323332

## Patient consent

**PatientInneneinverständniserklärung:**

Studienname: SWABIMS Follow up-Study

Datum/Version des Prüfplans: 21.03.2007

Datum/Version der Patienteninformation: 21.03.2007

Patientennummer: ...............

Hersteller der Prüfmedikation: Schering AG, Pfizer AG

Sponsoren: Neurologische Abteilung des Inselspitals Bern

Ich erteile meine Einwilligung, an dieser klinischen Studie mit Betaferon und Atorvastatin teilzunehmen. Ich hatte ausreichend Zeit, diese Entscheidung zu treffen, nachdem ich von dem unten angegebenen Arzt über Wesen, Bedeutung und Tragweite der Prüfung aufgeklärt worden bin. Ich bin bereit, den Anordnungen des Prüfarztes nachzukommen. Ich behalte mir das Rechts vor, meine Zustimmung jederzeit und ohne Angaben von Gründen zu widerrufen, ohne dass mir daraus Nachteile für meine weitere Behandlung entstehen. Ich bin mir bewusst, dass während der Studie die in der Patienteninformation genannten Anforderungen und Einschränkungen einzuhalten sind.

Ich bin einverstanden, dass die zuständigen Fachleute des Studienauftraggebers, der Behörden und der Kantonalen Ethikkomission Bern zu Prüf- und Kontrollzwecken in meine Originaldaten Einsicht nehmen dürfen, jedoch unter strikter Einhaltung der Vertraulichkeit.

Patient/Patientin:

____________________________ ___________________ ______________________

Name (Druckbuchstaben) Ort und Datum Unterschrift Patient/Patientin

Ich bestätige, dass der/die o.a. Patient/Patientin der Teilnahme dieser klinischen Studie zugestimmt hat. Über Art dieser Prüfung wurde er/sie vorher informiert.

Vermerke im Aufklärungsgespräch:

____________________________ _______________ ______________________

Name (Druckbuchstaben) Ort und Datum Unterschrift

Arzt/Ärztin,der/die
das Aufklärungsgespräch

durchgeführt hat
